# Pathogen profile of co-infections and mortality determinants among patients with severe fever with thrombocytopenia syndrome

**DOI:** 10.3389/fcimb.2025.1693567

**Published:** 2025-12-09

**Authors:** Yuan-Yuan Hu, Guo-Mei Xia, Han Zhang, Zhen-Hua Zhang

**Affiliations:** 1 Department of Otolaryngology-Head and Neck Surgery, The Second Affiliated Hospital of Anhui Medical University, Hefei, Anhui, China; 2 Department of Infectious Diseases, The Second Affiliated Hospital of Anhui Medical University, Hefei, Anhui, China; 3 Medical Records Department, The Second Affiliated Hospital of Anhui Medical University, Hefei, Anhui, China

**Keywords:** severe fever with thrombocytopenia syndrome, bacteria, fungi, co-infection, mortality

## Abstract

**Objectives:**

Severe fever with thrombocytopenia syndrome (SFTS) poses an increasing threat to global public health. This study aimed to investigate pathogen characteristics of bacterial/fungal co-infections and determine prognostic factors for mortality in co-infected SFTS patients.

**Methods:**

Demographic and clinical data were collected for eligible SFTS patients admitted to a sentinel hospital. LASSO and multivariable logistic regression were used to identify independent predictors.

**Results:**

A total of 629 SFTS patients were included, of whom 283 (45.0%) presented with co-infections. Most isolates (75.9%) obtained from respiratory specimens. Among 127 isolated fungi, *Aspergillus fumigatus* (29.9%) was predominant, followed by *Candida albicans* (28.3%). Of 159 bacteria, *Klebsiella pneumoniae* (13.8%) and *Acinetobacter baumannii* (13.8%) were the most common. Co-infected patients had significantly longer hospital stays, higher costs, and increased mortality. Multivariable logistic regression analysis revealed that antifungal use, transfer to the ICU, receiving CRRT, presence of central nervous system symptoms, mechanical ventilation, older age, and elevated levels of LDH were independent predictive factors of mortality risk among co-infected SFTS patients, and a nomogram was constructed. The ROC curve showed that the nomogram achieved an AUC of 0.949, and DCA confirmed the clinical utility.

**Conclusion:**

This study highlights that bacterial/fungal co-infections are highly prevalent and clinically impactful in SFTS patients, warranting enhanced microbial surveillance and targeted intervention.

## 1 Introduction

Severe fever with thrombocytopenia syndrome (SFTS), caused by Dabie bandavirus (genus Bandavirus, family Phenuiviridae, and order Bunyavirales), is a newly emerging tick-borne zoonotic infectious disease (Li et al., 2021). Since the first report in central China in 2009 and subsequent isolation of its pathogen, known as SFTS virus (SFTSV) at that time (Yu et al., 2011), SFTS has undergone rapid geographic expansion with reported cases in China, South Korea, and Japan (Li et al., 2021; Sharma and Kamthania, 2021; Cui et al., 2024; Charoensakulchai et al., 2025). Currently, SFTSV is the most widely used terminology for the pathogen (Luo et al., 2023). According to China’s national surveillance data from 2010 to 2023, there were 27,447 reported cases of SFTS, resulting in 1,326 deaths (Yue et al., 2024). The major clinical symptoms are characterized by fever, thrombocytopenia, leukocytopenia, and gastrointestinal symptoms, with an incubation period of one to two weeks (Yu et al., 2011). Severe cases can readily progress to multi-organ failure, with a high initial case mortality rate up to 30% (Yu et al., 2011). SFTS was listed as one of the top 10 serious infectious diseases requiring priority research and development by the World Health Organization (WHO) in 2017 (Mehand et al., 2018). Given its ongoing geographic expansion and high mortality burden, SFTS represents a growing global public health threat that demands immediate attention.

Co-infections with bacteria and fungi were commonly observed in viral infections and significantly associated with elevated mortality rates (Lee et al., 2022; Pham et al., 2024). Patients with SFTS likewise exhibited increased susceptibility to secondary infections (Xu et al., 2021; Ge et al., 2022; Song et al., 2023; Sun et al., 2025), though reported rates of bacterial/fungal co-infections varied considerably in the literature (Ge et al., 2022; Song et al., 2023; Sun et al., 2025). Evidence indicated that the mortality rates were significantly increased in SFTS patients with bacterial/fungal co-infections compared to those without co-infection (Song et al., 2023; Zuo et al., 2023). The pathogen complexity and overlapping symptomatology of co-infections often lead to diagnostic challenges and therapeutic delays in SFTS patients (Song et al., 2023). Therefore, accurately characterizing the clinical features and evaluating the impact of co-infections in SFTS patients is crucial for guiding early diagnosis and timely targeted antimicrobial therapy, thus enhancing patient survival.

At present, the pathogenesis of SFTS remains incompletely elucidated, and neither approved antiviral therapies nor licensed vaccines are available (Li et al., 2021; Luo et al., 2023). While numerous studies have established a series of epidemiological, clinical, and laboratory indicators associated with mortality in SFTS patients (He et al., 2020; Wang et al., 2022), the prognostic determinants for mortality risk in SFTS patients with bacterial/fungal co-infections remain unelucidated. Therefore, this study aims to evaluate the prevalence and pathogen characteristics of bacterial/fungal co-infections among SFTS patients based on admitted cases over a 7-year period at a sentinel hospital in Anhui Province, which represents a key SFTS-endemic area and ranks second nationwide in cumulative reported SFTS incidence in China (Yue et al., 2024). This study examined the impact of diverse co-infection patterns on clinical outcomes among co-infected SFTS patients. In addition, this study specifically investigated mortality-associated prognostic factors in co-infected SFTS patients to facilitate risk stratification and inform clinical decision-making for improved patient outcomes.

## 2 Materials and methods

### 2.1 Study design and participants

This retrospective study was conducted in a sentinel hospital for treating SFTS in Hefei, Anhui Province of China. The hospital is a tertiary teaching hospital with a capacity of 2,666 beds as of 2024, and was accredited as an Academic Medical Center Hospital by Joint Commission International (JCI) in January 2017. The study included first-time hospitalized patients diagnosed with SFTS between January 2019 and June 2025. Case definitions for SFTS adhered to the Guideline for the Prevention and Treatment of Severe Fever with Thrombocytopenia Syndrome (2010 version) promulgated by the original Ministry of Health of the People’s Republic of China (MHPRC) (Ministry of Health of the People’s Republic of China, 2011). The diagnosis of suspected cases was made on the basis of epidemiological history (history of working, living or traveling in mountainous areas or history of tick bite within the past 2 weeks preceding symptom onset), characteristic clinical manifestations (including fever, fatigue, nausea, and vomiting), and laboratory tests (such as decreased platelets and leukocytes in the peripheral blood). Confirmed cases were defined as those meeting the criteria for suspected cases along with at least one of the following laboratory-confirmed diagnostic indicators: 1) a positive nucleic acid test for SFTSV; 2) seroconversion to SFTSV-specific IgG or a ≥4-fold increase in IgG titers between acute and convalescent-phase; 3) a positive test for IgM against SFTVS in the acute phase; and 4) detection of SFTSV genome by next-generation sequencing (NGS).

Eligible participants met all of the following inclusion criteria: 1) confirmed cases of SFTS; 2) aged more than 18 years; and 3) availability of complete clinical diagnosis or microbiological data. The exclusion criteria included: 1) co-infection with other viral hemorrhagic fevers; and 2) history of hematological diseases, immunodeficiency diseases, or immunosuppression such as transplant procedures.

### 2.2 Data collection

A comprehensive dataset encompassing socio-demographic characteristics and clinical data was systematically extracted from electronic medical records for all enrolled patients including the following variables: 1) socio-demographics (age, sex, and occupation); 2) underlying conditions (hypertension, diabetes, coronary heart disease, and cerebral infarction); 3) epidemiological information and clinical manifestations [history of tick bite, date of onset, fever duration, highest body temperature (T_max_), chills, skin alterations (skin rashes, skin nodules, or changes in skin color), headache, fatigue, myalgia, cough/expectoration, chest distress, swollen lymph node, gastrointestinal symptoms (diarrhea, nausea, vomiting, poor appetite, or abdominal distension and pain), hemorrhage (gingival bleeding, petechia, ecchymosis, melena, or hemafecia), and central nervous system symptoms (muscle tremors, confusion, coma, convulsion, or other neurological signs)]; 4) laboratory parameters from the first blood collection at admission [viral loads (recorded as copies/mL and converted with log10 transformation), routine blood, biochemical, and coagulation markers]; 5) treatments received [transfer to the ICU, continuous renal replacement therapy (CRRT), central venous catheterization, mechanical ventilation, ribavirin, blood product, intravenous immune globulin, and antibacterial and antifungal use]; and 6) clinical outcomes [hospitalization costs, length of stay, and mortality (patients who survived within 28 days after admission were categorized into the survival group, while those who died were classified into the death group)]. Missing values were handled using the random forest-based missForest package (version 1.5) in R version 4.2.3, primarily for the following variables: SFTSV RNA (152 missing cases, 24.17%), calcium (139 missing cases, 22.10%), and procalcitonin (29 missing cases, 4.61%). The missingness proportions for these variables were consistent across the different outcome categories (death vs. survival), and no statistically significant differences were observed between the pre- and post-imputation values.

### 2.3 Definition of co-infections

Co-infections were defined as concurrent bacterial/fungal infections in SFTS patients. Bacterial/fungal infections were considered based on microbiological confirmation in symptomatic patients, excluding potential specimen contamination. Pathogen identification was confirmed through standardized microbiological methods including cultivation (sputum, bronchoalveolar lavage fluid, blood, urine, and sterile body fluid cultures), metagenomic next-generation sequencing (mNGS), fungal biomarker assays [ (Li et al., 2021; Cui et al., 2024)-*β*-D-glucan test (G test) and galactomannan test (GM test)]. The species identification of isolates was conducted using matrix-assisted laser desorption/ionization time-of-flight mass spectrometry (MALDI-TOF MS; Bruker Autoflex, Germany). Antimicrobial susceptibility testing and interpretation were performed following the guidelines established by the Clinical and Laboratory Standards Institute (CLSI) and the European Committee on Antimicrobial Susceptibility Testing (EUCAST) (Clinical and Laboratory Standards Institute (CLSI), 2025; The European Committee on Antimicrobial Susceptibility Testing (EUCAST), 2025).

### 2.4 Statistical analysis

Categorical variables were expressed as frequencies and percentages (%), with between-group comparisons performed using the chi-square (*χ*^2^) test. Normally distributed continuous variables were presented as means ± standard deviations (SDs) and difference comparisons were conducted using the independent samples *t*-test, while non-normally distributed variables were expressed as medians with interquartile ranges (IQR; P25, P75) and compared using Mann-Whitney U tests. Variables demonstrating statistically significant associations (*P* < 0.05) in univariate analyses were subsequently entered into a Least Absolute Shrinkage and Selection Operator (LASSO) regression model to automatically identify optimal predictors while controlling for overfitting. Finally, these candidate parameters selected through LASSO regression were subjected to the multivariable logistic regression analysis. Models selection was performed based on the minimum value of akaike information criterion (AIC). Risk estimates were expressed as adjusted odds ratios (AOR) accompanied by 95% confidence intervals (CIs). Additionally, significant variables (*P* < 0.05) were considered as independent predictive factors and incorporated into nomograms to facilitate individualized predictions. Model performance was comprehensively evaluated through receiver operating characteristic (ROC) curve analysis to assess discriminative ability and through decision curve analysis (DCA) to determine clinical utility. Statistical analyses were performed using SPSS version 26.0 (SPSS Inc., Chicago, IL, USA) and R version 4.2.3 (R Foundation for Statistical Computing, Vienna, Austria).

## 3 Results

### 3.1 Demographic and clinical features of SFTS patients

During the study period, 663 hospitalized patients were initially classified as suspected SFTS cases. Based on inclusion/exclusion criteria, 34 cases were excluded (31 lacked laboratory confirmation, 1 was dengue fever, 1 was scrub typhus, and 1 had an immunodeficiency). The final analysis included 629 confirmed SFTS cases, comprising 604 nucleic acid/NGS-positive and 25 IgM/IgG sero-positive cases (**Supplementary Table S1**). These participants had a median age of 68.00 (IQR 59.00-74.00; range 25.00-91.00) years, with males comprising 41.5%. Most participants (92.5%) were farmers. Tick bite exposure was reported by 149 patients (23.7%) prior to the onset of SFTS. The median duration between symptom onset and hospital admission was 5.00 (4.00-7.00) days. Fever (97.5%) was the predominant clinical manifestation, followed by gastrointestinal symptoms (94.8%) and fatigue (73.9%). Hypertension (23.4%) and diabetes (15.1%) represented the most prevalent underlying conditions. The median length of hospitalization was 12.00 (8.00-16.00) days, and the case mortality rate was 18.9% (119/629).

### 3.2 Co-infection and its impacts on outcomes of patients with SFTS

Among the 283 SFTS patients with co-infections, serological biomarker analysis demonstrated G test positivity in 71 cases, GM test positivity in 13 cases, and dual G/GM test positivity in 30 cases. Bacterial, fungal, and mixed bacterial-fungal infections were documented in 36 (5.7%), 175 (27.8%), and 72 (11.4%) cases, respectively (**Supplementary Table S1**). Microbiological culture isolated bacterial/fungal pathogens in 169 (59.7%) of 283 co-infected SFTS cases, comprising 111 monomicrobial and 58 polymicrobial infections. A total of 286 bacterial/fungal pathogens were isolated (**Supplementary Table S2**), with the majority (217 isolates) obtained from respiratory specimens, followed by blood cultures (45 isolates), urine cultures (14 isolates), stool cultures (9 isolates), and bone marrow culture (1 isolate). Among fungal isolates, *Aspergillus fumigatus* predominated (29.9%, 38/127), followed by *Candida albicans* (28.3%, 36/127), and *Aspergillus flavus* (13.4%, 17/127). The most prevalent bacterial pathogens were *Klebsiella pneumoniae* (13.8%, 22/159), *Acinetobacter baumannii* (13.8%, 22/159), and *Escherichia coli* (10.1%, 16/159). Notably, 20.1% (32/159) of bacterial isolates were identified as multidrug-resistant bacteria based on antimicrobial susceptibility testing.

Hospitalization duration was significantly longer in the co-infection group than in the control group (15.00 [IQR 10.00-20.00] days vs. 11.00 [8.00-13.00] days; *P* < 0.001). Hospitalization costs were higher in co-infected patients than non-co-infected patients (52,120.00 [37,281.00-74,681.00] RMB vs. 27,254.00 [10,293.00-42,209.00] RMB; *P* < 0.001). Similarly, mortality was also higher in co-infected patients than non-co-infected controls (23.7% vs. 15.0%; *P* = 0.006). When stratified by pathogen types, mortality rates were 22.2% for bacterial infections, 22.9% for fungal infections, and 26.4% for mixed bacterial-fungal infections.

### 3.3 Predictive factors for mortality risk among co-infected SFTS patients

**Figure 1:**
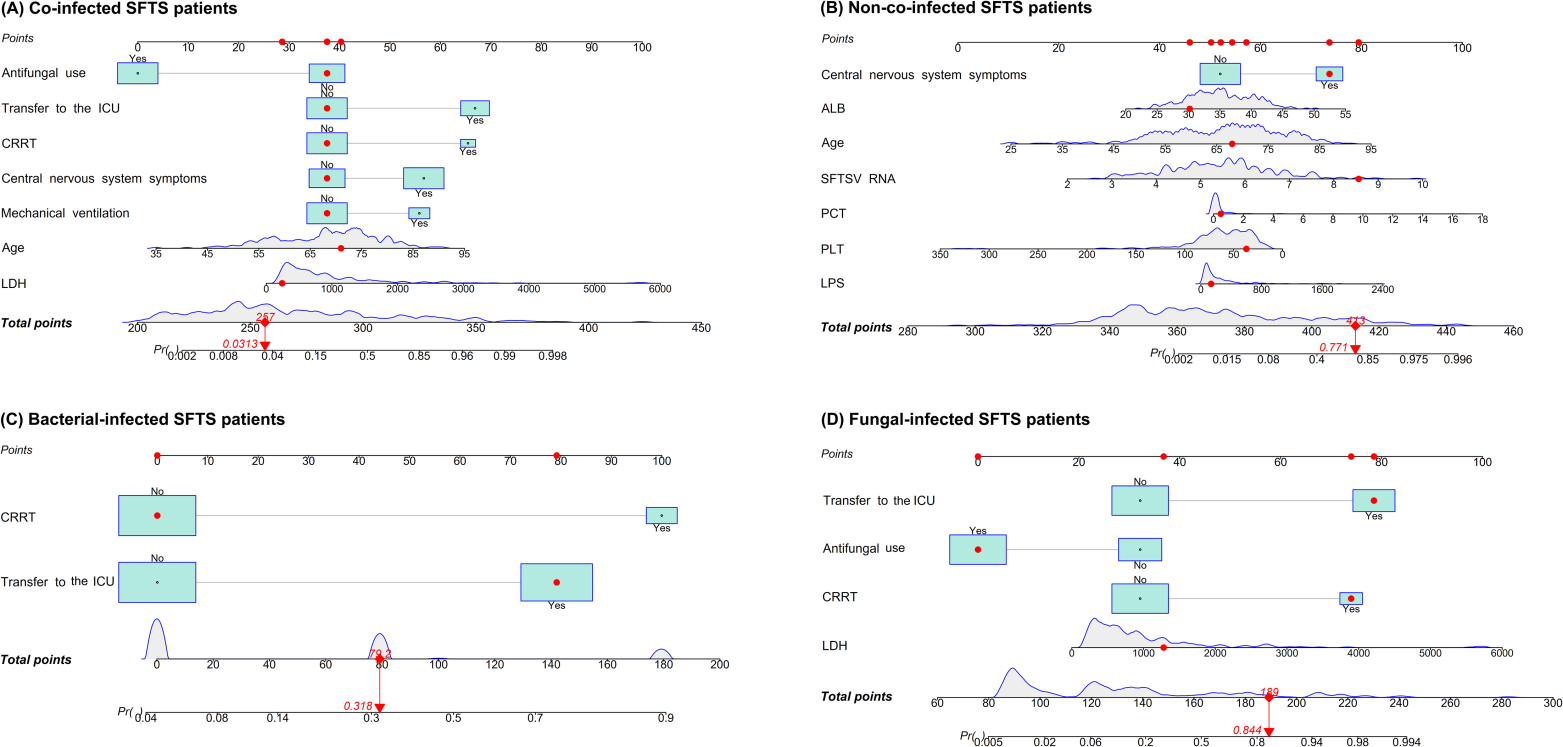
Nomograms for predicting mortality risk among co-infected SFTS patients **(A)**, non-co-infected SFTS patients **(B)**, bacterial-infected SFTS patients **(C)**, and fungal-infected SFTS patients **(D)**. Points were assigned to all predictors by projecting vertical lines to the 'Points' axis, followed by summation to derive the total points. The probability of outcomes progression was then determined based on the total points.

**Figure 2:**
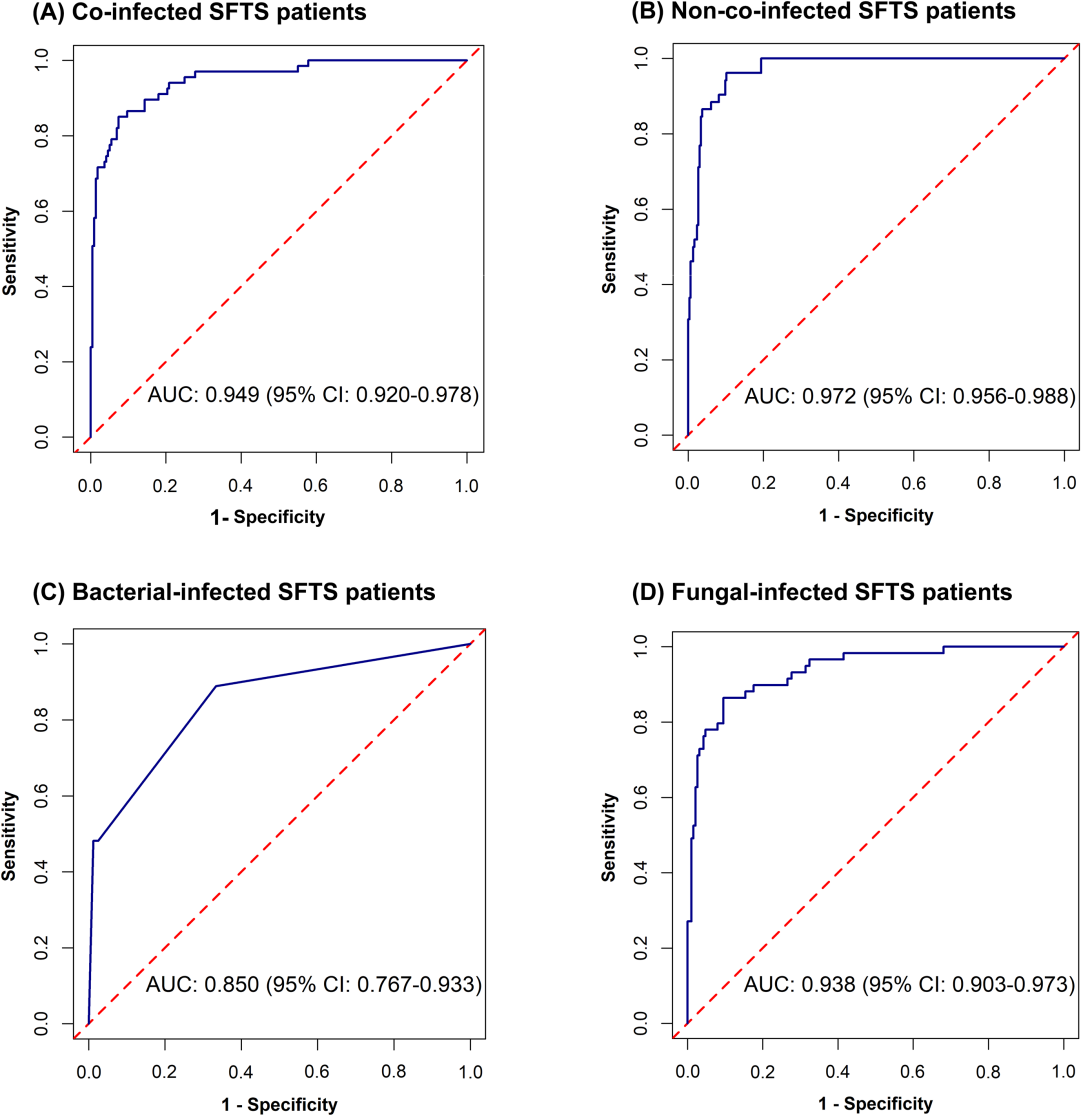
Receiver operating characteristic curves for predicting mortality risk among co-infected SFTS patients **(A)**, non-co-infected SFTS patients **(B)**, bacterial-infected SFTS patients **(C)**, and fungal-infected SFTS patients **(D)**.

**Figure 3:**
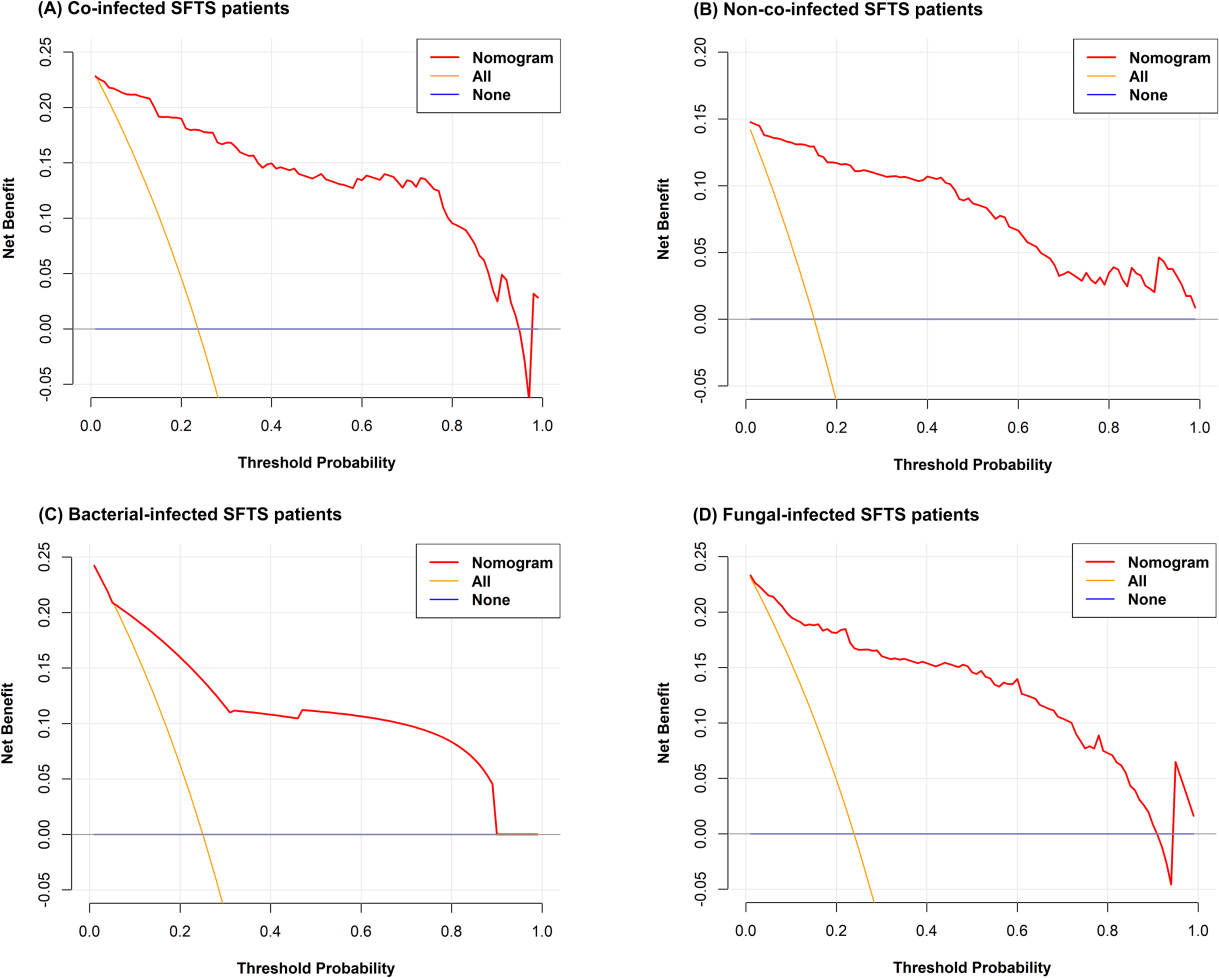
Decision curve analyses for predicting mortality risk among co-infected SFTS patients **(A)**, non-co-infected SFTS patients **(B)**, bacterial-infected SFTS patients **(C)**, and fungal-infected SFTS patients **(D)**.

Univariate analysis showed that differences were statistically significant in 32 variables between the death group and the survival group among co-infected SFTS patients (**Table 1**). Ten potential predictors including age, central nervous system symptoms, SFTSV RNA, lactate dehydrogenase (LDH), activated partial thromboplastin time (APTT), CRRT, transfer to the ICU, central venous catheterization, mechanical ventilation, and antifungal use were identified by LASSO regression based on the optimal λ_min_ value (λ=0.0048; **Supplementary Figure S1**). Subsequent multivariate logistic regression analysis revealed that antifungal use (AOR = 0.059, *P* < 0.001), transfer to the ICU (AOR = 9.190, *P* = 0.001), receiving CRRT (AOR = 8.271, *P* = 0.003), presence of central nervous system symptoms (AOR = 4.263, *P* = 0.028), mechanical ventilation (AOR = 3.988, *P* = 0.034), older age (AOR = 1.080, *P* = 0.005), and elevated levels of LDH (AOR = 1.001, *P* = 0.001) were independent predictive factors of mortality risk among co-infected SFTS patients based on the selected model (AIC: 146.39) (**Table 2**), and a prognostic nomogram was established (**Figure 1**). The ROC curve showed that the nomogram achieved an AUC of 0.949 (**Figure 2**). The DCA demonstrated sustained net benefit across an extended threshold probability range for the constructed nomogram (**Figure 3**).

**Table 1:** Comparison of socio-demographic and clinical data between the death and survival groups among 283 co-infected and 346 non-co-infected SFTS patients.

Variables	Co-infected SFTS patients			Non-co-infected SFTS patients		
	Death group (n=67)	Survival group (n=216)	*P*	Death group (n=52)	Survival group (n=294)	*P*
Demographic characteristics						
Age, years	73.00 (67.00-77.00)	68.00 (61.00-74.00)	0.008	75.50 (70.25-82.00)	65.00 (56.00-72.00)	<0.001
Farmer, n (%)	65 (97.0)	201 (93.1)	0.233	52 (100.0)	264 (89.8)	0.013
Male, n (%)	34 (50.7)	97 (44.9)	0.402	23 (44.2)	107 (36.4)	0.282
Underlying diseases						
Hypertension, n (%)	15 (22.4)	54 (25.0)	0.664	14 (26.9)	64 (21.8)	0.412
Diabetes, n (%)	10 (14.9)	41 (19.0)	0.450	10 (19.2)	434 (11.6)	0.126
Coronary heart disease, n (%)	1 (1.5)	11 (5.1)	0.201	1 (1.9)	7 (2.4)	1.000
Cerebral infarction, n (%)	5 (7.5)	15 (6.9)	0.885	3 (5.8)	16 (5.4)	1.000
Epidemiological information and clinical manifestations						
Tick bites history, n (%)	17 (25.4)	58 (26.9)	0.811	5 (9.6)	69 (23.5)	0.025
Time from onset to admission, days	5.00 (5.00-7.00)	5.00 (4.00-7.00)	0.380	4.00 (4.00-6.50)	5.00 (4.00-7.00)	0.134
Highest body temperature, ^o^C	39.10 (38.50-39.40)	39.00 (38.50-39.30)	0.015	38.80 (38.50-39.20)	38.95 (38.00-39.20)	0.331
Fever duration, days	9.00 (7.00-12.00)	8.00 (6.00-10.75)	0.018	7.00 (5.00-9.75)	6.00 (5.00-8.00)	0.039
Chills, n (%)	21 (31.3)	54 (25.0)	0.304	16 (30.8)	74 (25.2)	0.396
Skin alterations, n (%)	11 (16.4)	24 (11.1)	0.249	6 (11.5)	37 (12.6)	0.833
Headache, n (%)	6 (9.0)	38 (17.6)	0.088	1 (1.9)	54 (18.4)	0.003
Fatigue, n (%)	45 (67.2)	165 (76.4)	0.132	33 (63.5)	222 (75.5)	0.069
Myalgia, n (%)	17 (25.4)	66 (30.6)	0.416	12 (23.1)	95 (32.3)	0.184
Cough/expectoration, n (%)	23 (34.3)	122 (56.5)	0.002	16 (30.8)	58 (19.7)	0.073
Chest distress, n (%)	9 (13.4)	41 (19.0)	0.298	7 (13.5)	31 (10.5)	0.535
Swollen lymph node, n (%)	9 (13.4)	42 (19.4)	0.263	6 (11.5)	58 (19.7)	0.161
Gastrointestinal symptoms, n (%)	65 (97.0)	208 (96.3)	0.781	50 (96.2)	274 (93.2)	0.551
Hemorrhage, n (%)	43 (64.2)	124 (57.4)	0.325	27 (51.9)	94 (32.0)	0.005
Central nervous system symptoms, n (%)	62 (92.5)	96 (44.4)	<0.001	48 (92.3)	57 (19.4)	<0.001
Laboratory tests						
SFTSV RNA (lg, copies/mL)	6.92 (6.51-7.51)	5.93 (5.34-6.61)	<0.001	6.74 (6.08-7.49)	5.20 (4.51-5.91)	<0.001
PLT (×10^9^/L)	39.00 (26.00-52.00)	44.00 (33.00-63.50)	0.005	39.50 (30.00-59.75)	62.00 (43.75-79.25)	<0.001
WBC (×10^9^/L)	2.39 (1.59-3.94)	2.08 (1.45-3.83)	0.338	2.48 (1.61-4.44)	2.33 (1.45-3.88)	0.267
Neut (×10^9^/L)	1.74 (1.04-3.32)	1.48 (0.89-2.61)	0.158	1.87 (1.06-3.43)	1.18 (0.75-2.62)	0.006
Lymph (×10^9^/L)	0.43 (0.31-0.74)	0.48 (0.33-0.79)	0.346	0.48 (0.37-0.71)	0.61 (0.38-1.08)	0.045
RBC (×10^12^/L)	4.22 (3.88-4.56)	4.39 (3.94-4.73)	0.129	4.25 (3.91-4.70)	4.34 (3.97-4.65)	0.739
Hb (g/L)	127.00 (118.00-136.00)	131.00 (120.00-143.00)	0.088	126.00 (116.00-139.91)	128.50 (118.00-140.00)	0.506
CRP (mg/L)	6.10 (2.30-19.10)	3.55 (1.10-6.98)	0.001	4.55 (2.10-15.80)	2.10 (0.50-5.20)	<0.001
ALB (g/L)	32.21 ± 4.58	33.60 ± 5.31	0.053	33.48 ± 4.98	35.34 ± 5.04	0.014
Ca^2+^ (mmol/L)	1.86 (1.74-1.92)	1.92 (1.84-2.00)	<0.001	1.91 (1.84-1.98)	1.99 (1.91-2.06)	<0.001
Na^+^ (mmol/L)	135.40 (132.70-138.70)	134.60 (132.80-138.05)	0.331	135.55 (134.00-137.80)	135.90 (133.30-139.20)	0.435
K^+^ (mmol/L)	3.84 (3.44-4.38)	3.71 (3.37-4.07)	0.072	4.09 (3.78-4.44)	3.64 (3.37-4.00)	<0.001
AMY (U/L)	152.00 (76.00-250.99)	109.50 (76.75-159.75)	0.044	138.00 (96.25-202.25)	88.00 (65.00-126.00)	<0.001
LPS (U/L)	270.00 (127.00-592.00)	154.50 (86.25-337.25)	0.005	250.50 (116.00-554.75)	121.50 (71.75-265.25)	<0.001
CK (U/L)	728.00 (323.00-1979.00)	387.50 (171.00-1002.50)	<0.001	634.50 (204.50-1428.00)	252.50 (120.00-558.25)	<0.001
CKMB (U/L)	49.00 (30.00-98.00)	31.00 (23.00-48.75)	<0.001	39.50 (23.25-70.00)	26.00 (18.00-38.00)	<0.001
LDH (U/L)	992.00 (573.00-1824.00)	598.50 (361.00-971.50)	<0.001	843.00 (504.00-1282.00)	418.50 (316.00-630.00)	<0.001
ALT (U/L)	89.00 (55.00-143.00)	71.00 (43.00-129.00)	0.034	88.50 (41.00-139.75)	50.00 (30.00-96.25)	0.001
AST (U/L)	320.00 (174.00-515.00)	167.00 (88.00-336.50)	<0.001	238.50 (127.25-483.25)	103.50 (59.00-178.00)	<0.001
TBIL (umol/L)	9.50 (7.70-12.30)	8.70 (6.93-11.30)	0.084	9.35 (7.40-14.00)	8.80 (7.18-11.40)	0.147
CREA (umol/L)	91.00 (66.00-134.00)	72.50 (58.25-92.00)	<0.001	108.00 (77.75-136.00)	67.00 (53.00-85.00)	<0.001
ALP (U/L)	69.00 (56.00-91.00)	69.00 (58.25-89.00)	0.715	72.00 (56.75-96.69)	66.00 (55.75-81.25)	0.084
BUN (umol/L)	8.52 (6.20-14.37)	6.78 (5.35-8.89)	<0.001	10.67 (6.92-14.68)	5.66 (4.21-7.38)	<0.001
PCT (ng/mL)	0.35 (0.13-1.38)	0.19 (0.90-0.38)	<0.001	0.41 (0.18-1.69)	0.11 (0.06-0.22)	<0.001
PT (s)	12.00 (11.40-13.10)	11.40 (10.70-12.20)	<0.001	11.75 (11.13-12.95)	11.24 (10.50-12.00)	0.001
TT (s)	31.10 (23.90-51.90)	23.15 (20.00-28.85)	<0.001	26.05 (21.23-38.83)	20.85 (19.10-23.33)	<0.001
APTT (s)	46.00 (38.00-52.10)	37.50 (34.10-44.40)	<0.001	41.05 (35.83-50.98)	35.25 (31.90-40.03)	<0.001
D-D (ug/mL)	7.25 (2.96-17.64)	2.58 (1.39-5.98)	<0.001	5.64 (2.82-8.80)	2.01 (1.03-4.21)	<0.001
FIB (g/L)	2.04 (1.57-2.49)	2.30 (1.90-2.59)	0.002	2.19 (2.02-2.67)	2.44 (2.15-2.79)	0.027
Received treatment						
Transfer to the ICU, n (%)	56 (83.6)	39 (18.1)	<0.001	20 (38.5)	9 (3.1)	<0.001
CRRT, n (%)	30 (44.8)	4 (1.9)	<0.001	5 (9.6)	1 (0.3)	<0.001
Central venous catheterization, n (%)	45 (67.2)	18 (8.3)	<0.001	13 (25.0)	2 (0.7)	<0.001
Mechanical ventilation, n (%)	42 (62.7)	18 (8.3)	<0.001	11 (21.2)	0 (0.0)	<0.001
Blood product use, n (%)	39 (58.2)	65 (30.1)	<0.001	9 (17.3)	28 (9.5)	0.094
Ribavirin, n (%)	51 (76.1)	135 (62.5)	0.040	36 (69.2)	200 (68.0)	0.864
Systemic corticosteroid therapy	16 (23.9)	19 (8.8)	0.001	6 (11.5)	10 (3.4)	0.021
Intravenous immune globulin, n (%)	62 (92.5)	196 (90.7)	0.651	46 (88.5)	174 (59.2)	<0.001
Antibacterial use, n (%)	67 (100.0)	205 (94.9)	0.060	51 (98.1)	244 (83.0)	0.005
Antifungal use, n (%)	25 (37.3)	134 (62.0)	<0.001	7 (13.5)	15 (5.1)	0.032

SFTS, severe fever with thrombocytopenia syndrome; SFTSV, SFTS virus; WBC, white blood cell; PLT, platelet; Neut, neutrophil; Lymph, lymphocyte; RBC, red blood cell; Hb, hemoglobin; CRP, C-reactive protein; ALB, serum albumin; Ca^2^**^+^**, calcium; Na**^+^**, sodium; K**^+^**, potassium; AMY, amylase; LPS, lipase; CK, creatine phosphokinase; CKMB, creatine kinase-MB; LDH, lactate dehydrogenase; ALT, alanine aminotransferase; AST, aspartate aminotransferase; TBIL, total bilirubin; CREA, creatinine; ALP, alkaline phosphatase; BUN, blood urea nitrogen; PCT, procalcitonin; PT, prothrombin time; TT, thrombin time; APTT, activated partial thromboplastin time; D-D, D-dimer; FIB, fibrinogen; ICU, intensive care unit; CRRT, continuous renal replacement therapy.

**Table 2:** Multivariable logistic regression for risk factors of mortality among co-infected and non-co-infected SFTS patients.

Variables	*β*	Wald	AOR	95% CI	*P*
Co-infected SFTS patients					
Antifungal use	-2.837	22.778	0.059	0.018-0.188	<0.001
Transfer to the ICU	2.218	11.882	9.190	2.604-32.437	0.001
CRRT	2.113	8.817	8.271	2.051-33.361	0.003
Central nervous system symptoms	1.450	4.835	4.263	1.171-15.524	0.028
Mechanical ventilation	1.383	4.509	3.988	1.112-14.301	0.034
Age, years	0.077	8.056	1.080	1.024-1.139	0.005
LDH	0.001	10.485	1.001	1.000-1.002	0.001
Non-co-infected SFTS patients					
Central nervous system symptoms	2.828	16.317	16.912	4.288-66.702	<0.001
SFTSV RNA (lg, copies/mL)	1.021	12.248	2.775	1.567-4.915	<0.001
PCT	0.644	3.880	1.904	1.003-3.613	0.049
ALB	0.183	6.234	1.201	1.040-1.387	0.013
Age, years	0.157	18.063	1.170	1.088-1.258	<0.001
PLT	-0.036	5.527	0.965	0.936-0.994	0.019
LPS	0.002	5.732	1.002	1.000-1.004	0.017

SFTS, severe fever with thrombocytopenia syndrome; AOR, adjusted odds ratio; CI, confidence interval; ICU, intensive care unit; CRRT, continuous renal replacement therapy; LDH, lactate dehydrogenase; SFTSV, SFTS virus; PCT, procalcitonin; ALB, serum albumin; PLT, platelet; LPS, lipase.

Pathogen-stratified analyses using LASSO and multivariate logistic regression identified mortality predictors for bacterial and fungal co-infections (**Supplementary Figure S2**, **Table 3**). CRRT (bacterial co-infection: AOR = 18.371, *P* = 0.001; fungal co-infection: AOR = 9.503, *P* = 0.001) and transfer to the ICU (bacterial co-infection: AOR = 10.020, *P* = 0.001; fungal co-infection: AOR = 12.217, *P* < 0.001) were common independent predictors of mortality risk among SFTS patients with bacterial and fungal co-infections. The mortality risk model among SFTS patients with fungal co-infections additionally incorporated antifungal use (AOR = 0.102, *P* < 0.001) and LDH (AOR = 1.001, *P* = 0.001). The nomograms for both subgroups were constructed (**Figure 1**), and ROC and DCA curves are shown in **Figure 2** and **Figure 3**.

**Table 3:** Multivariable logistic regression for risk factors of mortality among co-infected SFTS patients stratified by pathogens (bacteria/fungi).

Variables	*β*	Wald	AOR	95% CI	*P*
Bacterial co-infection					
CRRT	2.911	11.407	18.371	3.393-99.473	0.001
Transfer to the ICU	2.305	10.940	10.020	2.557-39.260	0.001
Fungal co-infection					
Transfer to the ICU	2.503	17.295	12.217	3.756-39.740	<0.001
Antifungal use	-2.288	16.202	0.102	0.033-0.309	<0.001
CRRT	2.252	10.514	9.503	2.437-37.062	0.001
LDH	0.001	11.750	1.001	1.000-1.001	0.001

SFTS, severe fever with thrombocytopenia syndrome; AOR, adjusted odds ratio; CI, confidence interval; CRRT, continuous renal replacement therapy; ICU, intensive care unit; LDH, lactate dehydrogenase.

### 3.4 Predictive factors for mortality risk among SFTS patients without co-infection

Univariate analysis revealed that 38 variables differed significantly between the death and survival groups among SFTS patients without co-infection (**Table 1**). LASSO regression analysis refined these candidate variables based on the λ_min_ value (λ=0.011; **Supplementary Figure S1**), identifying 15 potential predictors including age, headache, central nervous system symptoms, SFTSV RNA, platelet (PLT), serum albumin (ALB), amylase (AMY), lipase (LPS), aspartate aminotransferase (AST), blood urea nitrogen (BUN), procalcitonin (PCT), mechanical ventilation, transfer to the ICU, CRRT, and systemic corticosteroid therapy. Multivariate logistic regression analysis demonstrated that presence of central nervous system symptoms (AOR = 16.912, *P* < 0.001), elevated levels of SFTSV RNA (AOR = 2.775, *P* < 0.001), elevated levels of PCT (AOR = 1.904, *P* = 0.049), elevated levels of ALB (AOR = 1.201, *P* = 0.013), older age (AOR = 1.170, *P* < 0.001), decreased levels of PLT (AOR = 0.965, *P* = 0.019), and elevated levels of LPS (AOR = 1.002, *P* = 0.017) were significantly associated with mortality risk among SFTS patients without co-infection (**Table 2**). Subsequently, a nomogram was developed based on the selected model (AIC: 110.55), wherein a higher total score corresponded to elevated mortality risk among SFTS patients without co-infection (**Figure 1**). The ROC curve analysis confirmed that the nomogram demonstrated discriminative ability with an AUC of 0.972 (**Figure 2**). Furthermore, DCA indicated a favorable net benefit across a broad range of threshold probabilities (**Figure 3**).

## 4 Discussion

This study revealed a 45.0% prevalence of bacterial/fungal co-infections among SFTS patients, demonstrating a substantial burden. This observed prevalence of bacterial/fungal co-infections either approximated or exceeded the 27.4-44.9% range reported in a few studies (Ge et al., 2022; Song et al., 2023; Sun et al., 2025), with discrepancies potentially stemming from differences in patient demographics, infection surveillance methodologies, or other study-specific confounding factors. However, the pathogenesis of SFTS remains incompletely characterized, particularly regarding the complex interplay between SFTSV and host immune responses (Yang et al., 2022). Immunomodulation to SFTSV involves a wide range of immune cells, cytokine networks, and signaling pathways (Yang et al., 2022). Notably, while the immune response can mediate SFTSV clearance, excessive immune activation may trigger pathological hyperinflammation, resulting in immunopathogenic damage (Yang et al., 2022). In severe SFTS cases, SFTSV can cause a devastating cytokine storm syndrome and immunosuppression (Luo et al., 2023). The immune dysregulation likely predisposes SFTS patients to secondary bacterial/fungal infections.

Consistent with prior studies (Zhang et al., 2022; Song et al., 2023; Song et al., 2023), this study demonstrated that bacterial/fungal co-infections in SFTS patients were significantly associated with the prolonged hospitalization duration, increased hospitalization costs, and higher mortality rates. These results position microbial co-infections as a pivotal prognostic factor for adverse clinical outcomes, emphasizing the imperative for early detection and targeted intervention for SFTS patients with co-infections. Notably, mixed bacterial-fungal infections showed an increased mortality rate (26.4%) compared to single-pathogen infections (bacterial: 22.2%; fungal: 22.9%), highlighting pathogen compositions as key modulators of mortality rates in co-infected SFTS cases. Supporting this observation, one prior study documented distinct mortality risks associated with diverse pathogens in SFTS patients (Ge et al., 2022), reinforcing the necessity of robust microbiological surveillance to optimize therapeutic strategies.

The present study, in alignment with an established literature (Ge et al., 2022), provides conclusive evidence that respiratory infections were predominant, warranting increased clinical attention. Fungal infections occurred more frequently than bacterial infections in SFTS patients, which is consistent with a previous report (Song et al., 2023). The predominant fungal pathogen isolated from co-infected SFTS patients was *Aspergillus fumigatus*, followed by *Candida albicans*. Invasive fungal infections usually occur in immunocompromised and critically ill patients, and pose a major clinical challenge characterized by high morbidity and mortality, compounded by diagnostic complexities (Trelles et al., 2025). Besides, a notable observation was that cultures exhibited constrained diagnostic yield in SFTS patients with suspected fungal infections. This highlights the essential role of multimodal diagnostic approaches, integrating serological assays, microscopic examination, and molecular techniques to facilitate timely and precise diagnosis (Zhang et al., 2022). Furthermore, this analysis also revealed *Klebsiella pneumoniae* and *Acinetobacter baumannii* as the predominant bacterial pathogens isolated from co-infected SFTS patients (Song et al., 2023; Sun et al., 2025). Notably, through large-scale sampling, this study has identified a significantly broader bacterial spectrum compared with prior studies (Song et al., 2023; Sun et al., 2025), suggesting greater microbial complexity in co-infected SFTS patients.

Currently, in the absence of effective targeted therapeutics or vaccines for SFTS, optimized supportive care remains the primary realistic strategy. This study provides novel insights into predictors of mortality risk in co-infected SFTS patients. The prognostic nomogram demonstrated excellent predictive accuracy (AUC = 0.949), potentially facilitating early risk stratification in clinical practice. Antifungal treatment was significantly associated with decreased mortality risk in co-infected SFTS patients, including the fungal co-infection subgroup. One previous study also revealed that antifungal therapy was related to improved survival in SFTS patients with community-acquired bacterial/fungal co-infections (Ge et al., 2022). Despite notable advances in diagnostic techniques and expanding options of antifungal agents, invasive fungal infections remain an escalating concern in the management of critically ill patients (Dimopoulos et al., 2013). This finding, together with prior evidence (Dimopoulos et al., 2013), demonstrates that the implementation of validated clinical prediction rules coupled with rapid diagnostic assays facilitates early identification of high-risk fungal-infected patients who are most likely to benefit from early therapeutic intervention. Notably, 90.1% and 28.8% of enrolled patients receiving antibacterial and antifungal treatment, respectively. The proportion of antibiotic prescribing considerably exceeded the confirmed cases of bacterial infections, potentially leading to excessive antibiotic utilization. Besides, this study revealed that co-infected SFTS patients showed remarkably higher ICU admission rates (33.6%) compared to non-co-infected patients (8.4%), with transfer to the ICU emerging as the independent mortality predictor both in the overall co-infected patient cohort and across the bacterial and fungal co-infection subgroups in multivariate analysis. Severe SFTS cases are characterized by rapid disease progression and high rates of complications such as multiple organ failure, with reported ICU mortality reaching 51.8% in a prior study (Liu et al., 2025). The current study found that co-infected patients requiring CRRT had a significantly higher mortality rate compared to those who did not. Renal dysfunction is one of multiple organ dysfunction syndromes of severe SFTS patients. This finding was consistent with one previous study demonstrating that acute kidney injury was associated with increased mortality in SFTS patients (Zhang et al., 2023). This study identified the significant associations between central nervous system symptoms and mortality risk in both co-infected and non-co-infected SFTS patients, it further corroborates that neurological manifestations serve as a significant prognostic indicator of mortality in SFTS patients (Song et al., 2022; Wang et al., 2022; Xia et al., 2024). It is noteworthy that mechanical ventilation emerged as a significant predictor of mortality risk in co-infected patients. Mechanical ventilation was independently associated with increased mortality risk in infected patients, likely representing a clinical marker of disease severity. Moreover, mechanical ventilation is a common invasive procedure that is frequently associated with bacterial/fungal infections (Ang and Sun, 2018; Thomas-Rüddel et al., 2022), which could further aggravate the clinical severity of SFTS. In the present study, co-infected SFTS patients exhibited a significantly higher median age (70.00 years) compared to non-co-infected patients (67.00 years). Advanced age was associated with an increased mortality risk, possibly due to comorbidities, reduced organ compensatory capacity, immune system dysregulation and excessive inflammatory responses, which might exacerbate disease severity (Li et al., 2022; Liang et al., 2023). The importance of LDH in SFTS progression was further validated based on findings from this study (He et al., 2021; Liu et al., 2024; Xu et al., 2025). LDH serves as a biomarker of cellular damage and tissue necrosis, with elevated serum LDH levels reflecting cytokine storms and multi-organ injury severity caused by SFTS (Xu et al., 2025). In contrast to its lack of association in co-infected patients, a lower platelet count was significantly associated with mortality in SFTS patients without co-infection. This divergence may suggest a shift in the underlying pathophysiology of fatal outcomes between these groups. In non-co-infected individuals, mortality appears primarily driven by the direct pathogenic effect of the virus, wherein thrombocytopenia serves as a key indicator of viral burden and disease severity, making it a significant predictor of mortality risk (Wang et al., 2022; Yan et al., 2025). Conversely, in co-infected patients, mortality is increasingly influenced by secondary bacterial or fungal infections, and the prognostic value of the isolated hematologic parameter like platelet count may be diminished.

Despite these findings advancing understanding of microbial etiology, clinical consequences, and mortality predictors of co-infected SFTS patients, several limitations should be acknowledged. First, the clinical compliance of microbiological specimen collection may not be absolutely guaranteed, which might have resulted in an underestimation of the co-infection incidence. Second, while this study was conducted at a sentinel hospital managing substantial SFTS cases located in a high-endemicity region, the generalizability of these findings to other epidemiological and clinical settings requires further validation. Third, the retrospective nature of this study inherently lacked serial biomarker measurements, thereby limiting temporal assessment of associations between predictive factors and outcome development.

This study demonstrates that bacterial/fungal co-infections occur frequently among SFTS patients and are associated with a substantially increased mortality risk. These findings support the implementation of enhanced microbial surveillance protocols for high-risk SFTS inpatients. Furthermore, multiple prognostic factors may elevate mortality risk in co-infected SFTS patients, highlighting the importance of early recognition and timely therapeutic intervention to improve patient care. Further research should focus on combining longitudinal cohort design with time-series laboratory monitoring to prospectively validate these predictive factors and refine predictive models among SFTS patients.

## Data Availability

The original contributions presented in the study are included in the article/**Supplementary Material**. Further inquiries can be directed to the corresponding author.
