# Supplementary material for: Pathogen profile of co-infections and mortality determinants among patients with severe fever with thrombocytopenia syndrome
Source: Front Cell Infect Microbiol. 2025 Dec 9;15:1693567. doi: 10.3389/fcimb.2025.1693567 (PMC12722868; doi:10.3389/fcimb.2025.1693567)
Supplement: Supplementary file 1 [file Table1.doc]

**Table S1 Distribution of SFTS laboratory-confirmed diagnostic indicators and co-infections**

| Patient ID | Sex | Age (year) | SFTS laboratory-confirmed diagnostic indicators | | | | Co-infections | | Death |
| --- | --- | --- | --- | --- | --- | --- | --- | --- | --- |
|  |  |  | SFTSV RNA | IgM | IgG | NGS | Fungal | Bacterial |  |
| 1 | Female | 55 | Positive | Positive |  |  |  |  | No |
| 2 | Female | 72 | Positive |  |  |  | Positive |  | No |
| 3 | Male | 68 |  |  |  | Positive | Positive |  | Yes |
| 4 | Male | 60 | Positive | Positive | Positive |  |  |  | No |
| 5 | Male | 75 | Positive |  |  |  | Positive |  | No |
| 6 | Female | 66 | Positive |  |  |  |  |  | No |
| 7 | Female | 70 | Positive |  |  |  | Positive | Positive | No |
| 8 | Female | 53 | Positive | Positive | Positive |  |  |  | No |
| 9 | Female | 50 | Positive | Positive | Positive |  |  |  | No |
| 10 | Female | 58 | Positive |  |  |  | Positive | Positive | Yes |
| 11 | Female | 61 | Positive | Positive |  |  | Positive |  | Yes |
| 12 | Male | 59 | Positive |  |  |  |  |  | Yes |
| 13 | Male | 37 | Positive |  |  |  |  |  | No |
| 14 | Female | 77 | Positive |  |  |  |  |  | No |
| 15 | Female | 70 | Positive |  |  |  | Positive | Positive | No |
| 16 | Female | 72 | Positive |  |  |  | Positive | Positive | No |
| 17 | Male | 74 | Positive | Positive | Positive |  | Positive |  | Yes |
| 18 | Male | 66 | Positive | Positive | Positive |  | Positive |  | No |
| 19 | Female | 59 | Positive | Positive |  |  |  |  | No |
| 20 | Female | 56 | Positive |  |  |  | Positive | Positive | No |
| 21 | Male | 64 | Positive |  |  |  |  |  | No |
| 22 | Female | 76 | Positive |  |  |  | Positive | Positive | No |
| 23 | Female | 52 | Positive |  |  |  | Positive | Positive | No |
| 24 | Female | 77 | Positive | Positive |  |  |  |  | No |
| 25 | Male | 75 | Positive |  |  |  |  |  | No |
| 26 | Female | 65 | Positive | Positive | Positive |  |  |  | No |
| 27 | Female | 81 | Positive |  |  |  |  |  | No |
| 28 | Female | 58 | Positive | Positive |  |  |  |  | No |
| 29 | Male | 75 | Positive |  |  |  | Positive |  | No |
| 30 | Female | 80 | Positive |  |  |  | Positive | Positive | Yes |
| 31 | Female | 59 | Positive | Positive |  |  |  |  | No |
| 32 | Female | 56 | Positive |  |  |  | Positive |  | No |
| 33 | Female | 67 | Positive |  |  |  |  |  | No |
| 34 | Female | 30 | Positive | Positive |  |  |  |  | No |
| 35 | Female | 85 | Positive | Positive |  |  |  |  | Yes |
| 36 | Female | 53 | Positive |  |  |  | Positive |  | No |
| 37 | Female | 68 | Positive |  |  |  |  |  | No |
| 38 | Male | 32 | Positive | Positive |  |  |  |  | No |
| 39 | Female | 56 | Positive | Positive | Positive |  |  |  | No |
| 40 | Male | 35 | Positive | Positive | Positive |  | Positive |  | No |
| 41 | Female | 72 | Positive | Positive |  |  | Positive |  | No |
| 42 | Female | 72 | Positive |  |  |  |  |  | No |
| 43 | Male | 77 | Positive |  |  |  | Positive |  | No |
| 44 | Female | 67 | Positive |  |  |  |  |  | Yes |
| 45 | Male | 59 | Positive |  |  |  | Positive |  | No |
| 46 | Male | 74 | Positive |  |  |  |  |  | No |
| 47 | Male | 59 | Positive | Positive | Positive |  | Positive |  | No |
| 48 | Female | 74 |  | Positive |  |  | Positive | Positive | Yes |
| 49 | Female | 62 | Positive |  |  |  | Positive |  | No |
| 50 | Male | 80 | Positive |  |  |  |  |  | Yes |
| 51 | Female | 73 | Positive | Positive |  |  |  |  | No |
| 52 | Female | 64 | Positive |  |  |  |  |  | No |
| 53 | Female | 71 | Positive | Positive | Positive |  | Positive |  | No |
| 54 | Male | 81 | Positive |  |  |  | Positive |  | No |
| 55 | Female | 77 | Positive |  |  |  | Positive |  | Yes |
| 56 | Female | 49 | Positive | Positive |  |  |  |  | No |
| 57 | Male | 73 | Positive |  |  |  | Positive | Positive | Yes |
| 58 | Male | 62 | Positive |  |  |  | Positive |  | No |
| 59 | Female | 65 | Positive |  |  |  |  |  | No |
| 60 | Female | 68 | Positive |  |  |  | Positive |  | No |
| 61 | Male | 58 | Positive |  |  |  | Positive |  | No |
| 62 | Female | 75 |  |  | Positive |  |  |  | Yes |
| 63 | Female | 71 | Positive |  |  |  | Positive | Positive | No |
| 64 | Female | 86 | Positive |  |  |  |  |  | Yes |
| 65 | Female | 72 | Positive |  |  |  |  |  | Yes |
| 66 | Male | 82 | Positive | Positive | Positive |  |  |  | No |
| 67 | Female | 58 | Positive |  |  |  |  |  | No |
| 68 | Female | 82 | Positive |  |  |  |  |  | Yes |
| 69 | Female | 69 | Positive |  |  |  |  |  | No |
| 70 | Male | 77 | Positive |  | Positive |  |  |  | Yes |
| 71 | Female | 53 | Positive |  |  |  |  |  | No |
| 72 | Female | 86 | Positive |  |  |  |  |  | Yes |
| 73 | Female | 69 | Positive |  |  |  |  |  | No |
| 74 | Female | 72 | Positive |  |  |  | Positive | Positive | No |
| 75 | Male | 71 | Positive | Positive | Positive |  |  |  | No |
| 76 | Female | 58 | Positive |  |  |  | Positive | Positive | No |
| 77 | Female | 66 | Positive |  |  |  | Positive |  | No |
| 78 | Male | 61 | Positive |  |  |  | Positive | Positive | No |
| 79 | Male | 71 | Positive |  |  |  |  |  | No |
| 80 | Female | 68 | Positive |  |  |  |  | Positive | No |
| 81 | Female | 61 | Positive |  |  |  | Positive |  | No |
| 82 | Female | 70 | Positive | Positive |  |  | Positive |  | No |
| 83 | Female | 58 | Positive |  |  |  |  |  | No |
| 84 | Female | 70 | Positive |  |  |  | Positive | Positive | Yes |
| 85 | Male | 54 | Positive |  |  |  |  | Positive | No |
| 86 | Male | 78 | Positive |  |  |  | Positive | Positive | No |
| 87 | Female | 76 | Positive |  |  |  | Positive |  | No |
| 88 | Female | 60 | Positive |  |  |  |  |  | No |
| 89 | Female | 71 | Positive | Positive | Positive |  |  |  | No |
| 90 | Male | 71 | Positive |  |  |  | Positive | Positive | No |
| 91 | Female | 83 | Positive |  |  |  | Positive |  | No |
| 92 | Female | 58 | Positive |  |  |  | Positive |  | Yes |
| 93 | Female | 68 | Positive |  |  |  |  | Positive | Yes |
| 94 | Female | 77 | Positive | Positive |  |  | Positive |  | No |
| 95 | Female | 62 | Positive |  |  |  |  |  | No |
| 96 | Male | 25 | Positive |  |  |  |  |  | No |
| 97 | Male | 69 | Positive |  |  |  | Positive | Positive | No |
| 98 | Male | 60 | Positive |  |  |  | Positive |  | Yes |
| 99 | Female | 72 | Positive | Positive | Positive |  |  |  | No |
| 100 | Male | 54 | Positive |  |  |  |  |  | No |
| 101 | Female | 78 | Positive |  |  |  | Positive | Positive | Yes |
| 102 | Female | 71 | Positive |  |  |  |  |  | Yes |
| 103 | Female | 53 | Positive |  |  |  |  |  | No |
| 104 | Female | 79 | Positive |  |  |  |  |  | No |
| 105 | Female | 57 | Positive |  |  |  | Positive |  | No |
| 106 | Female | 69 | Positive | Positive | Positive |  | Positive |  | No |
| 107 | Male | 68 | Positive |  |  |  | Positive |  | Yes |
| 108 | Female | 71 | Positive |  |  |  |  |  | No |
| 109 | Female | 69 | Positive |  |  |  |  |  | Yes |
| 110 | Male | 54 | Positive | Positive | Positive |  |  |  | No |
| 111 | Female | 81 | Positive |  |  |  | Positive |  | No |
| 112 | Female | 61 | Positive |  |  |  |  |  | No |
| 113 | Male | 61 | Positive | Positive | Positive |  | Positive |  | No |
| 114 | Female | 58 | Positive | Positive | Positive |  |  | Positive | No |
| 115 | Male | 82 | Positive |  |  |  | Positive |  | No |
| 116 | Male | 41 | Positive |  |  |  |  |  | No |
| 117 | Female | 70 | Positive | Positive | Positive |  |  |  | No |
| 118 | Male | 72 | Positive | Positive | Positive |  | Positive | Positive | Yes |
| 119 | Male | 69 | Positive |  |  |  |  |  | No |
| 120 | Male | 76 | Positive |  |  |  |  |  | Yes |
| 121 | Female | 55 | Positive | Positive | Positive |  |  |  | No |
| 122 | Female | 79 | Positive |  |  |  |  |  | No |
| 123 | Male | 69 | Positive |  |  |  |  |  | No |
| 124 | Male | 67 | Positive | Positive | Positive |  |  |  | No |
| 125 | Male | 71 | Positive | Positive |  |  | Positive |  | No |
| 126 | Male | 76 | Positive | Positive |  |  | Positive |  | No |
| 127 | Male | 66 | Positive |  |  |  |  |  | Yes |
| 128 | Male | 58 | Positive | Positive | Positive |  |  |  | No |
| 129 | Male | 90 | Positive |  |  |  | Positive | Positive | Yes |
| 130 | Male | 84 | Positive |  |  |  |  |  | No |
| 131 | Female | 50 | Positive |  |  |  | Positive |  | No |
| 132 | Female | 62 | Positive |  |  |  |  |  | No |
| 133 | Female | 73 | Positive |  |  |  |  |  | No |
| 134 | Male | 83 | Positive |  |  |  |  | Positive | Yes |
| 135 | Male | 75 | Positive | Positive | Positive |  | Positive | Positive | No |
| 136 | Female | 67 | Positive | Positive |  |  |  |  | No |
| 137 | Male | 77 | Positive |  |  |  | Positive | Positive | No |
| 138 | Female | 83 | Positive | Positive | Positive |  | Positive | Positive | No |
| 139 | Female | 91 | Positive |  |  |  |  |  | Yes |
| 140 | Female | 71 | Positive | Positive | Positive |  |  |  | No |
| 141 | Male | 66 | Positive | Positive | Positive |  | Positive |  | No |
| 142 | Female | 83 | Positive | Positive | Positive |  |  |  | No |
| 143 | Female | 81 | Positive | Positive | Positive |  | Positive |  | No |
| 144 | Male | 71 | Positive |  |  |  |  |  | No |
| 145 | Male | 73 | Positive | Positive |  |  |  |  | No |
| 146 | Male | 74 | Positive |  |  |  |  | Positive | No |
| 147 | Female | 80 | Positive | Positive |  |  |  |  | Yes |
| 148 | Female | 80 | Positive | Positive | Positive |  |  |  | No |
| 149 | Male | 58 | Positive | Positive |  |  |  |  | No |
| 150 | Male | 76 | Positive |  |  |  | Positive |  | No |
| 151 | Female | 89 | Positive |  |  |  | Positive |  | Yes |
| 152 | Male | 72 | Positive | Positive | Positive |  | Positive |  | No |
| 153 | Male | 58 | Positive | Positive | Positive |  | Positive |  | No |
| 154 | Female | 68 | Positive |  |  |  | Positive |  | No |
| 155 | Female | 51 | Positive | Positive | Positive |  |  |  | No |
| 156 | Female | 68 | Positive |  |  |  |  |  | No |
| 157 | Male | 62 | Positive |  |  |  |  |  | Yes |
| 158 | Male | 73 | Positive |  |  |  |  |  | No |
| 159 | Female | 78 | Positive | Positive | Positive |  | Positive | Positive | No |
| 160 | Female | 83 | Positive | Positive |  |  | Positive |  | Yes |
| 161 | Female | 58 | Positive | Positive |  |  |  |  | No |
| 162 | Male | 74 | Positive | Positive |  |  | Positive |  | No |
| 163 | Male | 64 | Positive | Positive | Positive |  | Positive | Positive | No |
| 164 | Female | 72 | Positive |  |  |  |  | Positive | Yes |
| 165 | Male | 56 | Positive | Positive |  |  | Positive |  | No |
| 166 | Male | 80 | Positive | Positive |  |  | Positive |  | No |
| 167 | Male | 61 | Positive |  |  |  |  |  | No |
| 168 | Female | 71 | Positive | Positive | Positive |  |  |  | No |
| 169 | Male | 67 | Positive | Positive | Positive |  | Positive |  | No |
| 170 | Male | 61 | Positive | Positive |  |  | Positive |  | No |
| 171 | Male | 48 | Positive | Positive |  |  |  | Positive | No |
| 172 | Female | 67 | Positive | Positive | Positive |  |  |  | No |
| 173 | Male | 55 | Positive | Positive |  |  |  |  | No |
| 174 | Male | 88 | Positive | Positive |  |  | Positive |  | No |
| 175 | Male | 54 | Positive |  |  |  | Positive |  | No |
| 176 | Male | 67 | Positive | Positive | Positive |  | Positive |  | No |
| 177 | Female | 86 | Positive | Positive |  |  | Positive |  | No |
| 178 | Male | 70 | Positive | Positive |  |  | Positive |  | No |
| 179 | Male | 74 | Positive |  |  |  | Positive |  | Yes |
| 180 | Male | 78 | Positive | Positive |  |  |  |  | No |
| 181 | Female | 58 | Positive |  |  |  |  |  | No |
| 182 | Female | 60 | Positive | Positive |  |  |  | Positive | No |
| 183 | Female | 50 | Positive | Positive |  |  |  | Positive | No |
| 184 | Female | 78 | Positive |  |  |  |  |  | No |
| 185 | Female | 83 | Positive | Positive |  |  |  |  | No |
| 186 | Female | 80 | Positive |  |  |  |  |  | No |
| 187 | Female | 80 | Positive |  |  |  | Positive |  | No |
| 188 | Female | 58 | Positive | Positive |  |  |  |  | No |
| 189 | Female | 91 | Positive | Positive |  |  | Positive |  | Yes |
| 190 | Female | 54 | Positive | Positive |  |  |  |  | No |
| 191 | Female | 46 | Positive | Positive |  |  |  |  | No |
| 192 | Female | 69 | Positive | Positive | Positive |  |  |  | No |
| 193 | Female | 60 | Positive |  |  |  |  |  | No |
| 194 | Female | 68 | Positive |  |  |  |  |  | No |
| 195 | Female | 67 | Positive | Positive |  |  | Positive | Positive | No |
| 196 | Male | 71 | Positive | Positive |  |  |  |  | No |
| 197 | Female | 79 | Positive | Positive |  |  | Positive |  | Yes |
| 198 | Female | 60 | Positive | Positive |  |  |  |  | No |
| 199 | Female | 67 | Positive |  |  |  |  |  | No |
| 200 | Female | 56 | Positive | Positive |  |  |  |  | No |
| 201 | Female | 60 |  | Positive | Positive |  |  |  | No |
| 202 | Female | 60 | Positive | Positive |  |  | Positive |  | No |
| 203 | Female | 73 | Positive | Positive |  |  | Positive |  | Yes |
| 204 | Male | 71 | Positive | Positive |  |  |  |  | No |
| 205 | Male | 73 | Positive | Positive |  |  | Positive |  | Yes |
| 206 | Female | 58 | Positive | Positive |  |  | Positive |  | No |
| 207 | Male | 75 | Positive |  |  |  |  |  | No |
| 208 | Female | 68 | Positive | Positive |  |  | Positive |  | No |
| 209 | Female | 74 | Positive | Positive |  |  |  |  | No |
| 210 | Female | 53 | Positive | Positive | Positive |  | Positive |  | No |
| 211 | Male | 80 | Positive |  |  |  | Positive |  | No |
| 212 | Male | 54 | Positive | Positive |  |  |  |  | No |
| 213 | Male | 73 | Positive | Positive |  |  |  |  | No |
| 214 | Female | 73 | Positive | Positive |  |  |  |  | No |
| 215 | Female | 68 | Positive | Positive |  |  |  |  | No |
| 216 | Male | 73 | Positive | Positive |  |  |  |  | No |
| 217 | Male | 74 | Positive |  |  |  | Positive |  | No |
| 218 | Female | 57 | Positive |  |  |  |  | Positive | No |
| 219 | Female | 56 | Positive |  |  |  |  | Positive | Yes |
| 220 | Male | 58 | Positive |  |  |  | Positive |  | Yes |
| 221 | Male | 73 | Positive |  |  |  | Positive |  | Yes |
| 222 | Female | 75 | Positive | Positive |  |  |  |  | No |
| 223 | Female | 80 | Positive | Positive |  |  | Positive |  | No |
| 224 | Female | 78 | Positive | Positive |  |  |  |  | No |
| 225 | Female | 78 | Positive |  |  |  | Positive | Positive | Yes |
| 226 | Male | 82 | Positive | Positive |  |  | Positive |  | Yes |
| 227 | Female | 67 | Positive |  |  |  | Positive | Positive | No |
| 228 | Female | 68 | Positive |  |  |  | Positive | Positive | No |
| 229 | Male | 70 | Positive |  |  |  | Positive |  | No |
| 230 | Male | 59 | Positive |  |  |  |  |  | No |
| 231 | Male | 85 | Positive |  |  |  |  |  | Yes |
| 232 | Female | 53 | Positive | Positive |  |  |  |  | No |
| 233 | Male | 82 | Positive | Positive |  |  |  |  | No |
| 234 | Male | 77 | Positive |  |  |  | Positive |  | Yes |
| 235 | Male | 48 | Positive |  |  |  |  |  | No |
| 236 | Female | 64 | Positive |  |  |  |  |  | No |
| 237 | Male | 80 | Positive |  |  |  | Positive |  | Yes |
| 238 | Male | 80 | Positive | Positive | Positive |  | Positive |  | No |
| 239 | Female | 53 | Positive | Positive |  |  |  |  | No |
| 240 | Male | 45 | Positive |  |  |  |  |  | No |
| 241 | Female | 72 | Positive | Positive |  |  |  |  | No |
| 242 | Male | 68 | Positive | Positive | Positive |  | Positive |  | No |
| 243 | Male | 69 | Positive | Positive | Positive |  |  |  | No |
| 244 | Female | 67 | Positive |  |  |  |  |  | No |
| 245 | Male | 61 | Positive | Positive |  |  |  |  | No |
| 246 | Male | 74 | Positive |  |  |  | Positive | Positive | Yes |
| 247 | Male | 60 | Positive | Positive |  |  |  |  | No |
| 248 | Male | 73 | Positive |  |  |  | Positive | Positive | Yes |
| 249 | Female | 49 | Positive | Positive | Positive |  |  |  | No |
| 250 | Female | 82 | Positive | Positive |  |  |  | Positive | No |
| 251 | Male | 76 | Positive | Positive |  |  | Positive |  | No |
| 252 | Female | 64 | Positive | Positive |  |  | Positive |  | No |
| 253 | Male | 59 | Positive |  |  |  | Positive |  | Yes |
| 254 | Female | 69 | Positive |  |  |  |  |  | No |
| 255 | Male | 69 | Positive | Positive |  |  | Positive |  | No |
| 256 | Male | 68 | Positive |  |  |  | Positive |  | No |
| 257 | Female | 47 | Positive | Positive |  |  | Positive |  | No |
| 258 | Male | 82 | Positive | Positive |  |  |  |  | Yes |
| 259 | Female | 68 | Positive |  |  |  | Positive | Positive | No |
| 260 | Female | 66 | Positive | Positive |  |  |  |  | Yes |
| 261 | Male | 68 | Positive | Positive |  |  |  |  | No |
| 262 | Male | 64 | Positive | Positive | Positive |  | Positive | Positive | No |
| 263 | Male | 71 | Positive | Positive |  |  |  |  | No |
| 264 | Male | 85 | Positive | Positive |  |  | Positive |  | No |
| 265 | Female | 65 | Positive |  |  |  |  |  | No |
| 266 | Male | 57 | Positive | Positive |  |  | Positive | Positive | No |
| 267 | Female | 52 | Positive | Positive |  |  |  |  | No |
| 268 | Female | 68 | Positive | Positive |  |  | Positive |  | No |
| 269 | Female | 71 | Positive | Positive | Positive |  | Positive | Positive | No |
| 270 | Female | 74 | Positive | Positive |  |  | Positive | Positive | No |
| 271 | Male | 77 | Positive | Positive |  |  | Positive | Positive | Yes |
| 272 | Female | 72 | Positive | Positive |  |  | Positive |  | No |
| 273 | Male | 52 | Positive | Positive |  |  |  |  | No |
| 274 | Female | 77 | Positive | Positive | Positive |  |  |  | No |
| 275 | Female | 63 | Positive | Positive |  |  |  |  | Yes |
| 276 | Male | 68 | Positive | Positive |  |  | Positive |  | No |
| 277 | Female | 68 | Positive | Positive | Positive |  |  |  | No |
| 278 | Female | 73 | Positive | Positive |  |  |  | Positive | No |
| 279 | Female | 69 | Positive | Positive |  |  |  |  | No |
| 280 | Female | 66 | Positive | Positive |  |  | Positive |  | No |
| 281 | Female | 55 | Positive |  |  |  |  |  | No |
| 282 | Male | 67 | Positive | Positive | Positive |  |  |  | No |
| 283 | Male | 66 | Positive |  |  |  |  |  | Yes |
| 284 | Female | 57 | Positive |  |  |  |  |  | No |
| 285 | Male | 63 | Positive |  |  |  | Positive | Positive | No |
| 286 | Male | 68 | Positive |  |  |  | Positive |  | Yes |
| 287 | Male | 76 | Positive | Positive |  |  |  |  | No |
| 288 | Male | 78 | Positive |  |  |  |  |  | Yes |
| 289 | Female | 58 | Positive |  |  |  | Positive |  | No |
| 290 | Female | 68 | Positive | Positive |  |  |  |  | No |
| 291 | Female | 75 | Positive | Positive |  |  | Positive |  | No |
| 292 | Male | 74 | Positive |  |  |  |  |  | Yes |
| 293 | Female | 73 | Positive | Positive |  |  | Positive |  | No |
| 294 | Female | 47 |  | Positive |  |  |  |  | No |
| 295 | Female | 71 | Positive |  |  |  | Positive | Positive | No |
| 296 | Female | 74 | Positive |  |  |  |  |  | No |
| 297 | Male | 67 | Positive |  |  |  |  |  | No |
| 298 | Male | 66 | Positive | Positive |  |  |  |  | Yes |
| 299 | Male | 56 | Positive |  |  |  |  |  | No |
| 300 | Male | 64 | Positive |  |  |  | Positive |  | No |
| 301 | Female | 82 | Positive | Positive |  |  |  |  | No |
| 302 | Female | 68 | Positive | Positive |  |  |  |  | No |
| 303 | Female | 55 | Positive | Positive |  |  |  |  | No |
| 304 | Female | 65 | Positive |  |  |  | Positive |  | No |
| 305 | Female | 75 | Positive |  |  |  |  | Positive | No |
| 306 | Male | 67 | Positive | Positive |  |  | Positive |  | No |
| 307 | Male | 51 | Positive | Positive |  |  | Positive |  | Yes |
| 308 | Female | 56 | Positive |  |  |  |  |  | No |
| 309 | Male | 76 | Positive | Positive |  |  |  |  | No |
| 310 | Female | 51 | Positive | Positive |  |  |  |  | No |
| 311 | Female | 80 | Positive | Positive | Positive |  | Positive | Positive | No |
| 312 | Female | 74 | Positive |  |  |  | Positive |  | No |
| 313 | Female | 70 | Positive |  |  |  | Positive |  | Yes |
| 314 | Female | 75 | Positive |  |  |  |  | Positive | Yes |
| 315 | Female | 56 | Positive | Positive | Positive |  | Positive | Positive | No |
| 316 | Male | 25 | Positive |  |  |  |  |  | No |
| 317 | Male | 64 | Positive |  |  |  |  |  | No |
| 318 | Female | 74 | Positive |  |  |  | Positive | Positive | No |
| 319 | Female | 55 | Positive | Positive |  |  |  |  | No |
| 320 | Female | 55 | Positive |  |  |  |  | Positive | No |
| 321 | Male | 70 | Positive |  |  |  |  |  | No |
| 322 | Male | 80 | Positive | Positive |  |  |  |  | No |
| 323 | Female | 59 | Positive | Positive |  |  |  |  | No |
| 324 | Female | 73 | Positive |  |  |  |  |  | No |
| 325 | Female | 75 | Positive | Positive |  |  |  |  | No |
| 326 | Male | 53 | Positive | Positive |  |  | Positive | Positive | No |
| 327 | Male | 80 | Positive | Positive | Positive |  | Positive |  | Yes |
| 328 | Male | 80 | Positive | Positive |  |  | Positive | Positive | No |
| 329 | Female | 51 | Positive | Positive |  |  |  |  | No |
| 330 | Female | 65 | Positive | Positive |  |  |  |  | No |
| 331 | Male | 64 | Positive |  |  |  |  |  | No |
| 332 | Female | 70 | Positive | Positive |  |  |  |  | No |
| 333 | Female | 53 | Positive |  |  |  |  |  | No |
| 334 | Male | 65 | Positive |  |  |  | Positive |  | No |
| 335 | Male | 65 | Positive | Positive |  |  | Positive | Positive | No |
| 336 | Male | 56 | Positive | Positive |  |  |  |  | No |
| 337 | Male | 70 | Positive |  |  |  |  |  | No |
| 338 | Female | 68 | Positive |  |  |  | Positive |  | No |
| 339 | Female | 59 | Positive | Positive | Positive |  |  |  | No |
| 340 | Female | 59 | Positive | Positive |  |  | Positive |  | No |
| 341 | Male | 69 | Positive |  |  |  | Positive | Positive | No |
| 342 | Male | 77 | Positive |  |  |  | Positive | Positive | Yes |
| 343 | Female | 77 | Positive |  |  |  |  |  | No |
| 344 | Male | 67 | Positive | Positive |  |  | Positive | Positive | Yes |
| 345 | Female | 73 | Positive |  |  |  | Positive |  | Yes |
| 346 | Female | 78 | Positive | Positive | Positive |  |  |  | No |
| 347 | Male | 53 | Positive | Positive | Positive |  | Positive |  | No |
| 348 | Female | 75 | Positive | Positive |  |  | Positive | Positive | Yes |
| 349 | Female | 50 | Positive | Positive |  |  |  |  | No |
| 350 | Female | 81 | Positive | Positive | Positive |  | Positive |  | No |
| 351 | Male | 73 | Positive |  |  |  | Positive | Positive | No |
| 352 | Male | 82 | Positive |  |  |  |  |  | No |
| 353 | Male | 65 | Positive | Positive |  |  | Positive | Positive | No |
| 354 | Male | 75 | Positive |  |  |  |  |  | Yes |
| 355 | Male | 49 | Positive |  |  |  | Positive |  | Yes |
| 356 | Female | 59 | Positive | Positive |  |  | Positive |  | No |
| 357 | Female | 66 | Positive |  |  |  |  |  | No |
| 358 | Female | 52 | Positive |  |  |  |  |  | No |
| 359 | Female | 63 | Positive | Positive |  |  | Positive |  | No |
| 360 | Male | 58 | Positive | Positive |  |  |  |  | No |
| 361 | Female | 56 | Positive | Positive |  |  |  |  | No |
| 362 | Male | 70 | Positive |  |  |  |  |  | No |
| 363 | Female | 74 | Positive | Positive |  |  | Positive |  | No |
| 364 | Male | 73 | Positive | Positive |  |  | Positive |  | No |
| 365 | Male | 84 | Positive | Positive |  |  |  |  | Yes |
| 366 | Female | 74 | Positive |  |  |  |  |  | Yes |
| 367 | Male | 68 | Positive | Positive |  |  | Positive | Positive | No |
| 368 | Male | 76 | Positive |  |  |  |  |  | Yes |
| 369 | Female | 70 | Positive | Positive | Positive |  | Positive | Positive | No |
| 370 | Female | 62 | Positive |  |  |  |  |  | No |
| 371 | Female | 79 | Positive |  |  |  |  |  | No |
| 372 | Male | 41 | Positive |  |  |  | Positive | Positive | No |
| 373 | Male | 59 | Positive |  |  |  |  |  | No |
| 374 | Male | 75 | Positive |  |  |  |  | Positive | Yes |
| 375 | Male | 46 | Positive | Positive |  |  |  | Positive | No |
| 376 | Male | 71 | Positive | Positive |  |  | Positive |  | No |
| 377 | Female | 82 | Positive | Positive |  |  |  |  | No |
| 378 | Male | 59 | Positive | Positive |  |  |  |  | No |
| 379 | Male | 80 | Positive | Positive |  |  | Positive |  | No |
| 380 | Female | 51 | Positive | Positive |  |  |  |  | No |
| 381 | Female | 63 | Positive |  |  |  | Positive |  | No |
| 382 | Male | 35 | Positive |  |  |  |  |  | No |
| 383 | Male | 80 | Positive |  |  |  |  |  | No |
| 384 | Female | 72 | Positive | Positive |  |  |  |  | No |
| 385 | Female | 73 | Positive | Positive |  |  |  |  | No |
| 386 | Male | 55 | Positive | Positive |  |  | Positive |  | No |
| 387 | Male | 58 | Positive | Positive |  |  |  |  | No |
| 388 | Female | 77 | Positive |  |  |  | Positive |  | No |
| 389 | Female | 62 | Positive | Positive |  |  | Positive |  | No |
| 390 | Male | 83 | Positive |  |  |  |  |  | Yes |
| 391 | Male | 73 | Positive |  |  |  | Positive | Positive | Yes |
| 392 | Male | 67 | Positive |  |  |  | Positive | Positive | Yes |
| 393 | Male | 73 | Positive | Positive |  |  |  |  | No |
| 394 | Female | 65 | Positive | Positive |  |  |  |  | No |
| 395 | Male | 60 | Positive |  |  |  |  |  | No |
| 396 | Male | 60 | Positive |  |  |  | Positive |  | No |
| 397 | Female | 74 | Positive | Positive |  |  | Positive |  | Yes |
| 398 | Female | 68 | Positive | Positive |  |  |  |  | No |
| 399 | Male | 72 | Positive |  |  |  | Positive |  | No |
| 400 | Female | 55 |  | Positive |  |  |  |  | No |
| 401 | Female | 53 | Positive |  |  |  | Positive |  | No |
| 402 | Male | 60 | Positive |  |  |  |  |  | No |
| 403 | Female | 68 | Positive |  |  |  |  | Positive | No |
| 404 | Male | 76 | Positive | Positive |  |  | Positive |  | No |
| 405 | Female | 73 | Positive | Positive |  |  | Positive |  | No |
| 406 | Female | 83 | Positive |  |  |  | Positive |  | Yes |
| 407 | Female | 71 | Positive | Positive |  |  | Positive |  | No |
| 408 | Male | 65 | Positive | Positive |  |  |  |  | No |
| 409 | Male | 71 | Positive |  |  |  |  |  | No |
| 410 | Female | 68 | Positive | Positive |  |  |  |  | No |
| 411 | Female | 71 | Positive |  |  |  |  | Positive | No |
| 412 | Male | 86 | Positive | Positive |  |  |  |  | Yes |
| 413 | Female | 70 | Positive |  |  |  |  |  | Yes |
| 414 | Male | 67 | Positive |  |  |  | Positive |  | No |
| 415 | Female | 68 | Positive |  |  |  |  |  | Yes |
| 416 | Male | 47 | Positive |  |  |  | Positive |  | No |
| 417 | Female | 53 | Positive |  |  |  | Positive |  | No |
| 418 | Female | 58 | Positive |  |  |  |  |  | No |
| 419 | Female | 77 | Positive |  |  |  |  |  | No |
| 420 | Female | 67 | Positive | Positive |  |  |  | Positive | No |
| 421 | Male | 73 | Positive | Positive |  |  |  |  | No |
| 422 | Male | 66 | Positive | Positive |  |  | Positive |  | No |
| 423 | Male | 72 | Positive |  |  |  | Positive | Positive | No |
| 424 | Male | 69 | Positive |  |  |  | Positive |  | No |
| 425 | Female | 56 | Positive | Positive |  |  | Positive |  | No |
| 426 | Female | 65 | Positive |  |  |  | Positive |  | No |
| 427 | Female | 80 | Positive | Positive |  |  |  |  | Yes |
| 428 | Female | 88 | Positive |  |  |  |  |  | Yes |
| 429 | Male | 64 | Positive |  |  |  |  |  | No |
| 430 | Male | 63 | Positive |  |  |  |  |  | No |
| 431 | Female | 74 | Positive |  |  |  |  |  | No |
| 432 | Female | 64 | Positive |  |  |  |  |  | No |
| 433 | Female | 63 | Positive | Positive |  |  |  |  | No |
| 434 | Male | 58 | Positive |  |  |  | Positive |  | Yes |
| 435 | Female | 75 | Positive |  |  |  | Positive |  | No |
| 436 | Female | 52 | Positive |  |  |  |  |  | No |
| 437 | Male | 80 | Positive |  |  |  |  | Positive | No |
| 438 | Male | 85 | Positive |  |  |  |  |  | Yes |
| 439 | Female | 69 |  | Positive |  |  |  |  | No |
| 440 | Female | 75 | Positive |  |  |  |  |  | Yes |
| 441 | Male | 69 |  | Positive |  |  |  |  | No |
| 442 | Male | 80 |  | Positive |  |  |  |  | No |
| 443 | Female | 55 | Positive | Positive |  |  | Positive |  | No |
| 444 | Male | 45 | Positive |  |  |  |  |  | No |
| 445 | Female | 75 | Positive |  |  |  |  | Positive | No |
| 446 | Female | 75 | Positive |  |  |  |  | Positive | No |
| 447 | Female | 60 | Positive |  |  |  |  |  | No |
| 448 | Female | 66 | Positive |  |  |  |  |  | No |
| 449 | Male | 70 | Positive |  |  |  |  |  | No |
| 450 | Female | 76 | Positive | Positive |  |  |  |  | No |
| 451 | Female | 80 | Positive |  |  |  |  |  | Yes |
| 452 | Female | 67 | Positive |  |  |  | Positive |  | Yes |
| 453 | Female | 70 | Positive | Positive |  |  |  |  | No |
| 454 | Female | 40 | Positive | Positive |  |  |  |  | No |
| 455 | Male | 63 | Positive |  |  |  |  |  | No |
| 456 | Female | 56 |  | Positive |  |  |  |  | No |
| 457 | Male | 69 | Positive | Positive |  |  |  |  | No |
| 458 | Male | 74 | Positive |  |  |  |  |  | Yes |
| 459 | Male | 75 | Positive |  |  |  | Positive |  | Yes |
| 460 | Female | 57 | Positive |  |  |  |  |  | No |
| 461 | Female | 52 | Positive |  |  |  |  |  | No |
| 462 | Female | 70 | Positive |  |  |  | Positive |  | Yes |
| 463 | Female | 60 | Positive | Positive |  |  |  |  | No |
| 464 | Female | 80 | Positive |  |  |  |  |  | No |
| 465 | Female | 69 | Positive |  |  |  | Positive |  | Yes |
| 466 | Female | 49 | Positive | Positive |  |  |  |  | No |
| 467 | Male | 50 | Positive | Positive |  |  |  | Positive | Yes |
| 468 | Male | 50 | Positive | Positive |  |  |  |  | No |
| 469 | Male | 71 | Positive | Positive |  |  |  |  | No |
| 470 | Female | 53 | Positive |  |  |  |  |  | No |
| 471 | Male | 82 | Positive |  |  |  |  |  | Yes |
| 472 | Female | 51 | Positive |  |  |  |  |  | No |
| 473 | Male | 66 | Positive |  |  |  |  |  | No |
| 474 | Female | 72 | Positive |  |  |  |  |  | No |
| 475 | Male | 67 | Positive |  |  |  |  |  | No |
| 476 | Female | 51 |  | Positive |  |  |  |  | No |
| 477 | Male | 58 | Positive | Positive |  |  |  |  | No |
| 478 | Male | 66 | Positive | Positive | Positive |  |  |  | No |
| 479 | Female | 64 | Positive |  |  |  |  |  | No |
| 480 | Female | 82 | Positive |  |  |  |  |  | Yes |
| 481 | Male | 84 | Positive |  |  |  | Positive |  | Yes |
| 482 | Female | 81 | Positive |  |  |  |  |  | No |
| 483 | Male | 73 | Positive |  |  |  |  |  | No |
| 484 | Male | 74 | Positive |  |  |  | Positive | Positive | No |
| 485 | Female | 73 |  | Positive |  |  |  |  | No |
| 486 | Female | 54 | Positive |  |  |  |  |  | No |
| 487 | Female | 54 | Positive |  |  |  |  |  | No |
| 488 | Female | 73 | Positive |  |  |  |  |  | Yes |
| 489 | Male | 56 | Positive |  |  |  | Positive |  | Yes |
| 490 | Male | 77 |  | Positive |  |  |  |  | No |
| 491 | Male | 35 |  | Positive |  |  |  |  | No |
| 492 | Female | 51 | Positive |  |  |  |  |  | No |
| 493 | Female | 74 | Positive | Positive |  |  |  | Positive | No |
| 494 | Male | 70 | Positive |  |  |  | Positive |  | Yes |
| 495 | Male | 70 | Positive |  |  |  |  |  | No |
| 496 | Male | 72 | Positive |  |  |  |  |  | Yes |
| 497 | Female | 57 | Positive |  |  |  |  |  | No |
| 498 | Female | 79 | Positive | Positive |  |  | Positive |  | No |
| 499 | Female | 69 | Positive |  |  |  |  |  | No |
| 500 | Male | 52 | Positive | Positive |  |  |  |  | No |
| 501 | Male | 54 | Positive |  |  |  | Positive |  | No |
| 502 | Male | 85 | Positive | Positive |  |  | Positive |  | No |
| 503 | Male | 68 | Positive |  |  |  | Positive |  | No |
| 504 | Male | 69 | Positive |  |  |  |  |  | No |
| 505 | Female | 60 | Positive |  |  |  |  |  | No |
| 506 | Female | 66 | Positive | Positive |  |  |  |  | No |
| 507 | Male | 73 | Positive |  |  |  | Positive | Positive | No |
| 508 | Female | 80 | Positive |  |  |  |  |  | No |
| 509 | Female | 56 |  | Positive |  |  |  |  | No |
| 510 | Male | 73 | Positive |  |  |  |  |  | No |
| 511 | Female | 68 |  | Positive |  |  |  |  | No |
| 512 | Female | 57 | Positive |  |  |  | Positive |  | Yes |
| 513 | Female | 55 | Positive | Positive |  |  |  | Positive | Yes |
| 514 | Female | 61 |  | Positive |  |  |  |  | No |
| 515 | Female | 63 |  | Positive |  |  |  | Positive | No |
| 516 | Female | 72 | Positive |  |  |  |  |  | Yes |
| 517 | Female | 73 |  | Positive |  |  |  |  | No |
| 518 | Female | 49 | Positive | Positive |  |  |  |  | No |
| 519 | Female | 52 | Positive | Positive |  |  | Positive | Positive | No |
| 520 | Female | 55 |  | Positive |  |  |  |  | No |
| 521 | Female | 62 |  | Positive |  |  | Positive |  | No |
| 522 | Male | 48 | Positive |  |  |  |  |  | No |
| 523 | Male | 65 |  | Positive |  |  |  |  | No |
| 524 | Male | 66 |  | Positive |  |  |  |  | No |
| 525 | Male | 71 | Positive |  |  |  |  |  | No |
| 526 | Female | 70 | Positive | Positive |  |  | Positive |  | No |
| 527 | Female | 57 |  | Positive |  |  |  | Positive | No |
| 528 | Female | 65 |  | Positive |  |  |  |  | No |
| 529 | Male | 63 | Positive | Positive |  |  | Positive | Positive | No |
| 530 | Male | 66 | Positive |  |  |  |  |  | No |
| 531 | Female | 67 | Positive | Positive |  |  |  |  | Yes |
| 532 | Male | 69 | Positive | Positive |  |  | Positive | Positive | No |
| 533 | Female | 78 | Positive | Positive |  |  |  |  | No |
| 534 | Male | 71 | Positive |  |  |  |  | Positive | No |
| 535 | Female | 60 | Positive |  |  |  |  |  | No |
| 536 | Female | 62 | Positive | Positive |  |  |  |  | No |
| 537 | Female | 53 | Positive |  |  |  |  |  | No |
| 538 | Female | 51 | Positive |  |  |  |  |  | No |
| 539 | Female | 57 |  | Positive |  |  |  |  | No |
| 540 | Female | 54 | Positive |  |  |  |  | Positive | No |
| 541 | Female | 66 | Positive | Positive |  |  | Positive |  | No |
| 542 | Male | 64 | Positive | Positive |  |  | Positive | Positive | No |
| 543 | Female | 69 | Positive |  |  |  | Positive |  | No |
| 544 | Female | 67 | Positive |  |  |  |  |  | No |
| 545 | Female | 63 | Positive |  |  |  |  |  | No |
| 546 | Female | 83 | Positive | Positive | Positive |  |  |  | Yes |
| 547 | Male | 67 | Positive | Positive |  |  |  |  | No |
| 548 | Female | 35 | Positive |  |  |  |  |  | No |
| 549 | Female | 81 | Positive | Positive | Positive |  |  |  | No |
| 550 | Female | 45 | Positive | Positive | Positive |  |  |  | No |
| 551 | Female | 54 | Positive |  |  |  |  | Positive | No |
| 552 | Female | 52 | Positive |  |  |  |  | Positive | No |
| 553 | Male | 75 | Positive |  |  |  | Positive |  | No |
| 554 | Female | 76 | Positive |  |  |  |  |  | Yes |
| 555 | Female | 62 | Positive |  |  |  |  |  | No |
| 556 | Female | 70 | Positive |  |  |  | Positive |  | No |
| 557 | Female | 59 | Positive |  |  |  |  |  | No |
| 558 | Female | 73 | Positive | Positive | Positive |  | Positive |  | No |
| 559 | Female | 66 | Positive | Positive | Positive |  | Positive | Positive | No |
| 560 | Female | 63 | Positive |  |  |  | Positive |  | Yes |
| 561 | Female | 74 | Positive |  |  |  |  |  | No |
| 562 | Female | 56 | Positive |  |  |  |  |  | No |
| 563 | Male | 53 | Positive |  |  |  |  |  | No |
| 564 | Male | 40 | Positive |  |  |  |  |  | No |
| 565 | Female | 82 | Positive |  |  |  | Positive |  | No |
| 566 | Female | 72 | Positive |  |  |  |  |  | No |
| 567 | Female | 53 | Positive |  |  |  |  |  | No |
| 568 | Female | 75 | Positive | Positive | Positive |  |  |  | Yes |
| 569 | Male | 60 | Positive |  |  |  | Positive | Positive | No |
| 570 | Female | 62 | Positive |  |  |  | Positive | Positive | No |
| 571 | Female | 63 | Positive | Positive |  |  | Positive |  | No |
| 572 | Male | 81 | Positive |  |  |  |  |  | Yes |
| 573 | Female | 75 | Positive |  |  |  | Positive | Positive | Yes |
| 574 | Female | 68 | Positive | Positive | Positive |  |  |  | No |
| 575 | Male | 71 | Positive | Positive | Positive |  |  |  | No |
| 576 | Male | 89 | Positive |  |  |  |  |  | Yes |
| 577 | Female | 77 | Positive |  |  |  |  |  | No |
| 578 | Male | 45 | Positive |  |  |  | Positive |  | No |
| 579 | Female | 76 | Positive | Positive |  |  | Positive |  | No |
| 580 | Female | 61 | Positive |  |  |  |  |  | No |
| 581 | Female | 80 | Positive | Positive | Positive |  |  |  | No |
| 582 | Female | 74 | Positive |  |  |  |  |  | Yes |
| 583 | Male | 70 | Positive |  |  |  | Positive |  | No |
| 584 | Male | 38 | Positive | Positive |  |  |  |  | No |
| 585 | Female | 74 | Positive |  |  |  | Positive |  | Yes |
| 586 | Male | 72 | Positive |  |  |  | Positive | Positive | No |
| 587 | Female | 62 | Positive |  |  |  |  |  | No |
| 588 | Female | 68 | Positive | Positive | Positive |  | Positive |  | No |
| 589 | Female | 61 | Positive |  |  |  |  |  | No |
| 590 | Female | 83 | Positive |  |  |  | Positive |  | Yes |
| 591 | Male | 86 | Positive |  |  |  |  |  | No |
| 592 | Male | 77 | Positive |  |  |  |  |  | No |
| 593 | Male | 58 | Positive |  |  |  |  | Positive | No |
| 594 | Female | 72 | Positive |  |  |  |  |  | No |
| 595 | Male | 62 | Positive |  |  |  | Positive |  | No |
| 596 | Female | 73 | Positive |  |  |  | Positive |  | No |
| 597 | Male | 57 | Positive |  |  |  |  |  | No |
| 598 | Female | 68 | Positive |  |  |  |  |  | No |
| 599 | Female | 56 | Positive |  |  |  | Positive |  | No |
| 600 | Female | 62 | Positive |  |  |  |  |  | No |
| 601 | Male | 80 | Positive | Positive |  |  | Positive |  | No |
| 602 | Male | 55 | Positive | Positive |  |  |  |  | No |
| 603 | Male | 78 | Positive |  |  |  | Positive |  | No |
| 604 | Female | 80 | Positive | Positive |  |  |  |  | No |
| 605 | Male | 77 | Positive |  |  |  | Positive |  | No |
| 606 | Female | 66 | Positive |  |  |  |  |  | No |
| 607 | Female | 70 | Positive |  |  |  | Positive |  | No |
| 608 | Female | 57 | Positive |  |  |  | Positive | Positive | No |
| 609 | Male | 54 | Positive |  |  |  |  |  | No |
| 610 | Female | 56 | Positive |  |  |  | Positive |  | No |
| 611 | Male | 62 | Positive |  |  |  |  |  | No |
| 612 | Male | 74 | Positive |  |  |  |  |  | No |
| 613 | Female | 69 | Positive |  |  |  |  | Positive | No |
| 614 | Female | 69 | Positive |  |  |  |  |  | No |
| 615 | Male | 65 | Positive |  |  |  | Positive |  | No |
| 616 | Female | 70 | Positive |  |  |  |  |  | Yes |
| 617 | Female | 76 | Positive |  |  |  |  |  | No |
| 618 | Female | 53 | Positive |  |  |  |  |  | No |
| 619 | Female | 60 | Positive |  |  |  | Positive | Positive | Yes |
| 620 | Female | 77 | Positive |  |  |  | Positive |  | No |
| 621 | Male | 58 | Positive |  |  |  |  |  | No |
| 622 | Female | 78 | Positive |  |  |  | Positive |  | No |
| 623 | Female | 78 | Positive |  |  |  | Positive |  | No |
| 624 | Male | 74 | Positive |  |  |  |  |  | No |
| 625 | Female | 80 | Positive |  |  |  |  |  | No |
| 626 | Male | 72 | Positive |  |  |  | Positive | Positive | No |
| 627 | Female | 62 | Positive |  |  |  |  |  | No |
| 628 | Female | 79 | Positive |  |  |  |  |  | No |
| 629 | Female | 85 | Positive |  |  |  | Positive |  | No |

**Table S2** Bacterial and fungal pathogens among co-infected SFTS patients

| Bacterial pathogens | | |  | Fungal pathogens | |
| --- | --- | --- | --- | --- | --- |
| Species distribution | n | Multidrug-resistant |  | Species distribution | n |
| **Sputum/bronchoalveolar lavage fluid** |  |  |  | **Sputum/bronchoalveolar lavage fluid** |  |
| *Klebsiella pneumoniae* | 21 | 5 |  | *Aspergillus fumigatus* | 36 |
| *Acinetobacter baumannii* | 20 | 12 |  | *Candida albicans* | 34 |
| *Stenotrophomonas maltophilia* | 10 | 0 |  | *Aspergillus flavus* | 17 |
| *Pseudomonas aeruginosa* | 9 | 2 |  | *Candida parapsilosis* | 4 |
| *Escherichia coli* | 9 | 3 |  | *Candida krusei* | 3 |
| *Enterobacter cloacae* | 5 | 1 |  | *Aspergillus niger* | 3 |
| *Staphylococcus aureus* | 5 | 3 |  | *Candida tropicalis* | 2 |
| *Burkholderia cepacia* | 4 | 0 |  | *Alternaria* | 2 |
| *Haemophilus influenzae* | 3 | 0 |  | *Candida guilliermondii* | 1 |
| *Chryseobacterium indologenes* | 2 | 0 |  | *Rhizopus arrhizus* | 1 |
| *Achromobacter xylosoxidans* | 2 | 0 |  | *Candida rugosa* | 1 |
| *Citrobacter freundii* | 2 | 0 |  | [*Trichosporon japonicum*](https://www.microbiologyresearch.org/content/journal/ijsem/10.1099/00207713-48-4-1425) | 1 |
| *Bacillus cereus* | 1 | 0 |  | *Trichophyton rubrum* | 1 |
| *Proteus mirabilis* | 1 | 0 |  | *Rhizopus microsporus* | 1 |
| *Enterobacter asburiae* | 1 | 0 |  | *Candida lusitaniae* | 1 |
| *Burkholderia ambifaria* | 1 | 0 |  | *Candida glabrata* | 1 |
| *Klebsiella oxytoca* | 1 | 1 |  | *Aspergillus ustus* | 1 |
| *Raoultella planticola* | 1 | 0 |  | *Aspergillus nidulans* | 1 |
| *Enterobacter asburiae* | 1 | 0 |  | *Aspergillus terreus* | 1 |
| *Pseudomonas putida* | 1 | 0 |  | *Schizophyllum commune* | 1 |
| *Acinetobacter pittii* | 1 | 0 |  | *Aspergillus versicolor* | 1 |
| *Enterobacter hormaechei* | 1 | 0 |  | **Blood** |  |
| *Burkholderia gladioli* | 1 | 0 |  | *Aspergillus fumigatus* | 1 |
| **Blood** |  |  |  | *Candida tropicalis* | 1 |
| *Staphylococcus epidermidis* | 9 | 0 |  | **Urine** |  |
| *Staphylococcus hominis* | 9 | 0 |  | *Candida krusei* | 1 |
| *Staphylococcus haemolyticus* | 4 | 0 |  | *Trichosporon asahii* | 1 |
| *Escherichia coli* | 3 | 0 |  | **Stool** |  |
| *Streptococcus mitis* | 2 | 0 |  | *Candida krusei* | 3 |
| *Streptococcus oralis* | 2 | 0 |  | *Candida glabrata* | 2 |
| *Acinetobacter baumannii* | 2 | 1 |  | *Candida albicans* | 2 |
| *Bacillus megaterium* | 1 | 0 |  | *Candida parapsilosis* | 1 |
| *Micrococcus luteus* | 1 | 0 |  | *Aspergillus fumigatus* | 1 |
| *Proteus mirabilis* | 1 | 0 |  |  |  |
| *Enterococcus faecalis* | 1 | 0 |  |  |  |
| *Klebsiella pneumoniae* | 1 | 0 |  |  |  |
| *Pseudomonas aeruginosa* | 1 | 0 |  |  |  |
| *Staphylococcus aureus* | 1 | 0 |  |  |  |
| *Corynebacterium striatum* | 1 | 0 |  |  |  |
| *Enterobacter cloacae* | 1 | 1 |  |  |  |
| *Enterococcus faecium* | 1 | 0 |  |  |  |
| *Staphylococcus capitis* | 1 | 0 |  |  |  |
| *Streptococcus pneumoniae* | 1 | 0 |  |  |  |
| **Urine** |  |  |  |  |  |
| *Escherichia coli* | 4 | 1 |  |  |  |
| *Enterococcus faecium* | 2 | 0 |  |  |  |
| *Enterococcus faecalis* | 1 | 0 |  |  |  |
| *Aeromonas caviae* | 1 | 0 |  |  |  |
| *Enterobacter cloacae* | 1 | 1 |  |  |  |
| *Pseudomonas putida* | 1 | 0 |  |  |  |
| *Staphylococcus capitis* | 1 | 0 |  |  |  |
| *Pseudomonas aeruginosa* | 1 | 1 |  |  |  |
| **Bone marrow** |  |  |  |  |  |
| *Staphylococcus epidermidis* | 1 | 0 |  |  |  |

SFTS, severe fever with thrombocytopenia syndrome.


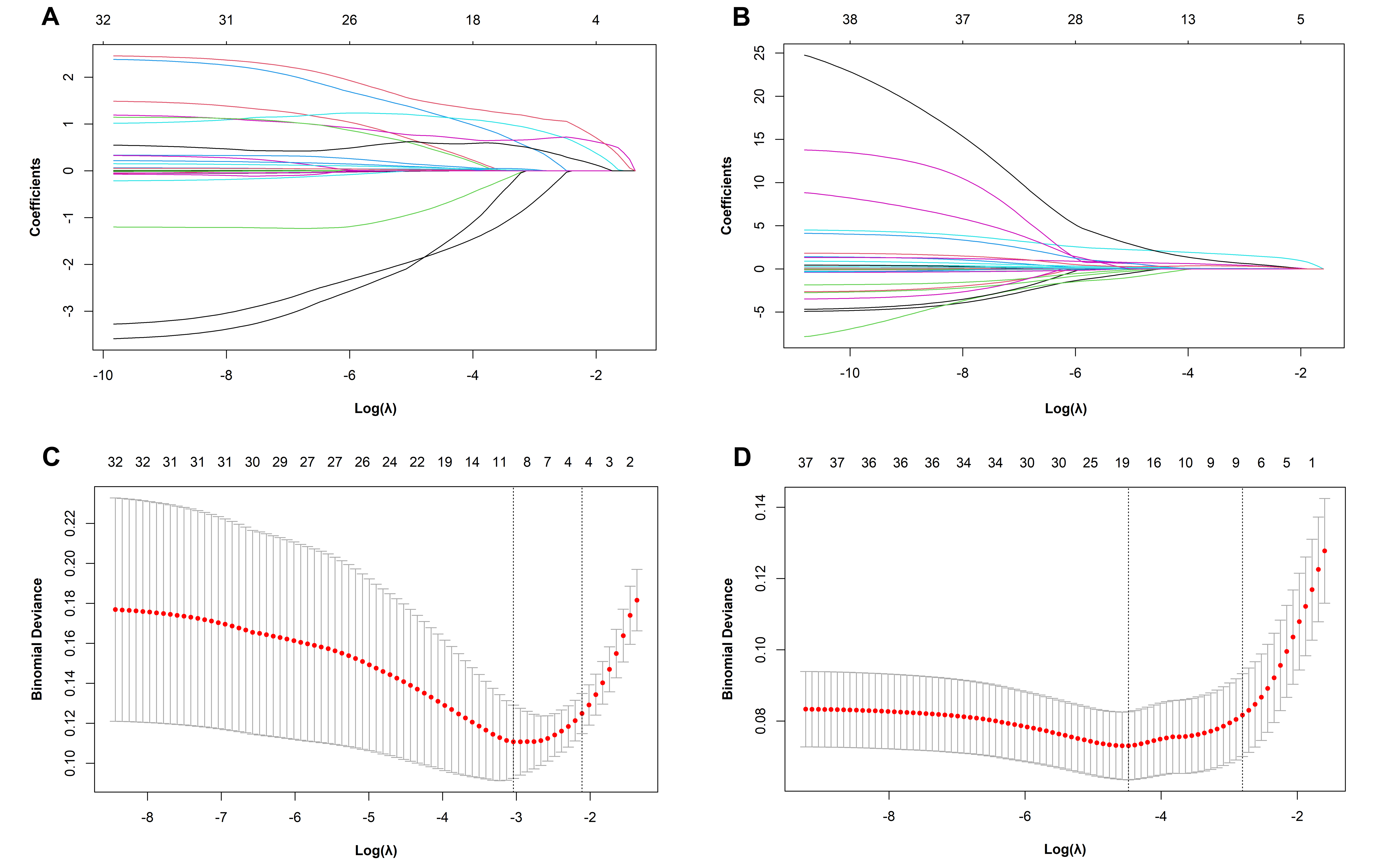


**Figure S1** Coefficient path plot (**A**) and cross-validation curve (**C**) for mortality risk among co-infected SFTS patients by LASSO regression. Coefficient path plot (**B**) and cross-validation curve (**D**) for mortality risk among non-co-infected SFTS patients by LASSO regression.


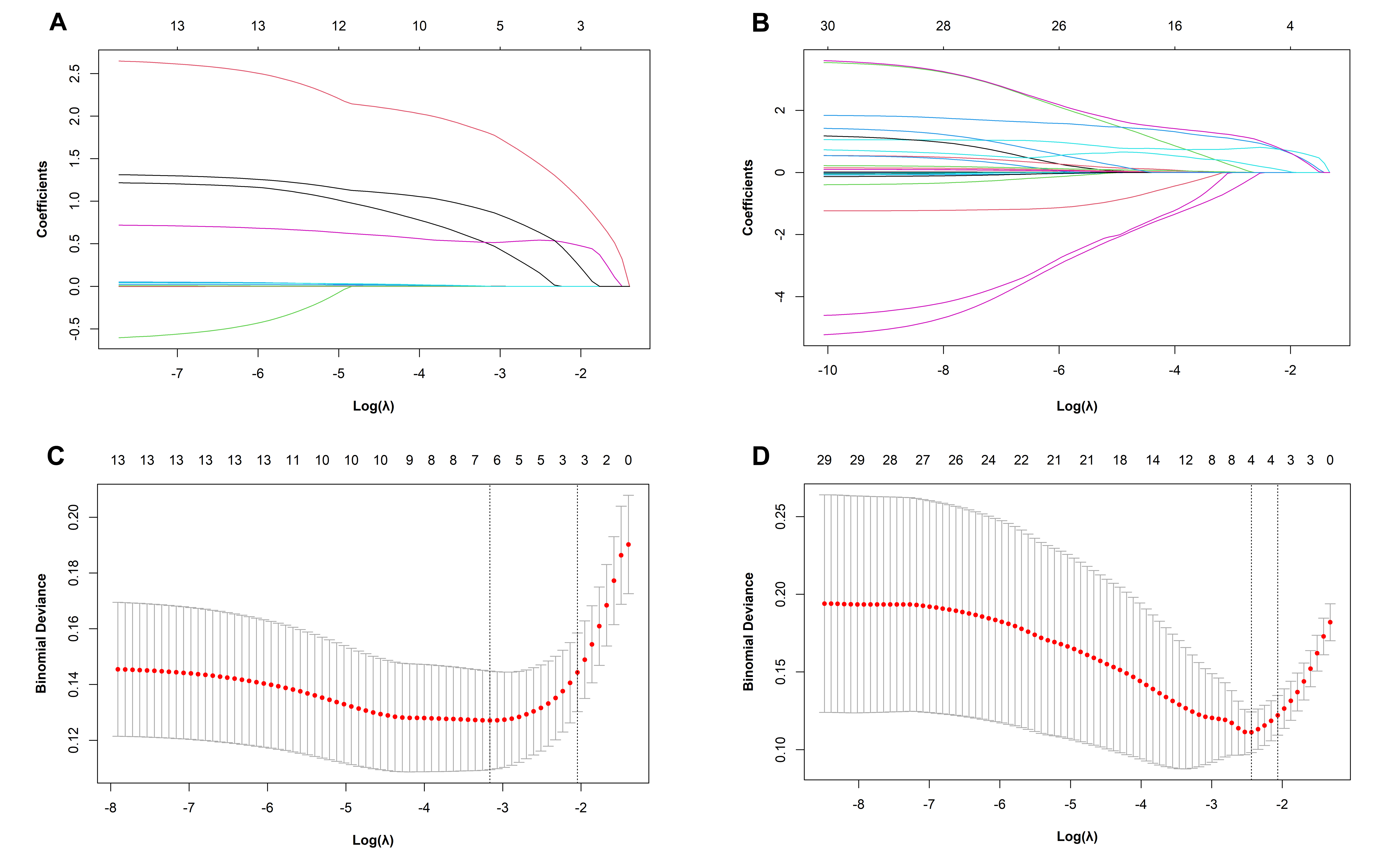


**Figure S2** Coefficient path plot (**A**) and cross-validation curve (**C**) for mortality risk among SFTS patients with bacterial co-infections by LASSO regression. Coefficient path plot (**B**) and cross-validation curve (**D**) for mortality risk among SFTS patients with fungal co-infections by LASSO regression
